# Protocol for Energy-Efficiency in Networked Control Systems Based on WSN

**DOI:** 10.3390/s18082590

**Published:** 2018-08-07

**Authors:** Jonathan M. Palma, Cristian Duran-Faundez, Leonardo de P. Carvalho, Cecília F. Morais, Ricardo C. L. F. Oliveira, Ernesto Rubio, Krzysztof Herman

**Affiliations:** 1 School of Electrical and Computer Engineering, University of Campinas—UNICAMP Campinas, 13083-852 São Paulo, Brazil; cfmorais@dt.fee.unicamp.br (C.F.M.); ricfow@dt.fee.unicamp.br (R.C.L.F.O.); 2 Department of Electrical and Electronic Engineering, University of the Bío-Bío, Concepción 4081112, Chile; aerubio@ubiobio.cl (E.R.); kherman@ubiobio.cl (K.H.); 3 Laboratório de Automação e Controle, Universidade de São Paulo—USP, 05508-900 São Paulo, Brazil; carvalho.lp@usp.br

**Keywords:** networkedcontrol systems, Wireless Sensor Networks, Hop-by-Hop transport scheme, semi-reliable communication protocol, packet loss

## Abstract

This paper proposes a new communication protocol for output-feedback control through multi-hop Wireless Sensor Network (WSN). The protocol is based on a Hop-by-Hop transport scheme and is especially devised to simultaneously fulfill two conflicting criteria: the network energy consumption and the stability/performance (in terms of $H_{\infty }$ norm) of the closed-loop system. The proposed protocol can be implemented by means of three heuristics, basically using distinct rules to control the maximum number of retransmissions allowed in terms of the voltage level of the batteries of the network nodes. As another contribution, a Markov jump based representation is proposed to model the packet loss in the communication channel, giving rise to a systematic procedure to determine the transition probability matrix and the Markov chain operation modes of a network with multiple information sources. The synthesis of the output-feedback controller is made in two steps (observer filter plus a state-feedback controller) for the Markov model assuming partial availability of the operation modes. The efficiency and applicability of the communication protocol is illustrated by means of a numerical experiment, based on a physical model of a coupled tanks plant. The features of each heuristic of implementation of the proposed protocol are presented in the numerical comparisons.

## 1. Introduction

Currently, the implementation of control techniques for dynamic systems through digital communication networks assumes an ideal communication channel, where the existence of data loss or delays is neglected. This assumption is possible thanks to high-performance state-of-the-art industrial networks such as ControlNet or DeviceNet [1]. However, the use of networking technologies with lower Quality of Service (QoS) in systems driven by classical controllers may bring significant drawbacks to ensure performance and stability [2,3,4,5]. Besides, other issues like coverage range, energetic autonomy and channel use [4,6,7,8,9,10,11] also must be taken into account by the designer. Despite all these difficulties, in recent years there has been increasing interest from the industrial sector as the technology can act as an alternative to wireless connect sensors, actuators and controllers; this provides benefits such as flexibility, monetary savings, ease of installation and mobility to connect wireless sensors and actuators (most unreachable with wired networks).

In accordance with industrial requirements for wireless communication technologies, which involve several economical and practical constraints, many applications adopt Wireless Sensor Networks (WSN) technologies [5] for connecting sensors and controllers, instead of using (relatively) high QoS wireless infrastructures such as those based on the IEEE 802.11 standards. WSNs are most based on the IEEE 802.15.4 standard, which was conceived for Low-Rate Wireless Personal Area Networks (LR-WPANs). Devices implementing those standards are known for being very resource-constrained (in processing/memorization capabilities, radio power output, etc.), in order to get as much energy savings as possible. Clearly, the economy of resources brings a set of problems that must be solved to assure high QoS levels, as required by classical control techniques.

To address the problems associated to regular WSN used as communication channel in closed-loop control, networked control systems (NCS) theory arises as an appeling control framework, allowing to obtain theoretical performance certificates even in presence of phenomena inherent to this kind of application, as packet loss and delay, as discussed in [2,3,12,13]. However, the available researches do not incorporate heuristics at the logical level aiming energy savings requirements, assuming that both channel use and the packet loss process are time-invariant and are not conditioned by external events.

The search for mechanisms for improving energy efficiency in the transmission of data in WSN is a transversal issue, but heuristics for minimizing energy costs in NCS are not abound. The work in [14] proposes a heuristic intending to provide energy-savings, but it assumes full-reliability in the communication protocol (property, which in practice, is not simple to guarantee).

Motivated by the aforementioned facts, the main contribution of this paper is the proposition of a communication protocol for Multi-Hop WSN suitable to deal with control applications, where two conflicting criteria must be fulfilled simultaneously: the minimization of the energy consumption and the stability and performance of the closed-loop system. Note that the latter can only be assured in full-reliable communication networks (no packet loss, implying in an unlimited number of retransmissions) and, clearly, is an unrealistic assumption in WSN scenario (low QoS). The particular performance criterion to be investigated is the $H_{\infty }$ norm, that is a well known robustness criterion associated to the attenuation of external disturbances, but other criteria could be considered as well. The proposed protocol is based on a Hop-by-Hop transport scheme and can be implemented by means of three distinct heuristics. The first one, called *Trade-off* in the context of dynamic systems, is an extension of the results originally proposed in [6] (which treat the design of filters in communication networks) to cope with control in semi-reliable networks. The solution proposed in [6] consists in choosing a single and finite number of retransmissions for all the nodes of the network, providing resources saving while, at the same time, assuring an $H_{\infty }$ performance suitable to the application. By limiting the maximum number of retransmissions, the expected value of global transmissions is reduced when compared with a full-reliable communication. As a consequence, it is possible to obtain a reduction in the network congestion, the average delay time of packet transmission, among other meaningful parameters. Differently from [6], that handles problems associated with the transmission of a single packet in a single path, this paper generalizes the approach to deal with the transmission of an arbitrary number of packets in several paths. The second heuristic is an adaptation of the methodology presented in [15] (that deals with another research area: image transmission in WSN) to cope with control of dynamical systems through communication networks. In [15], the authors proposed to modify the network reliability (the maximum number of retransmissions) by considering the status (particularly the battery charge) of the intermediate nodes in the path over a Hop-by-Hop sensor network. In the NCS context, subject of investigation of this paper, this method is adapted to start from a full-reliable communication (unlimited maximum number of retransmissions) and, gradually, to achieve a semi-reliable network by limiting the number of retransmissions of each intermediate node. Finally, a third heuristic combining some features of the previous two is an original proposition of this paper. Differently from the second, in this new heuristic the network always operates in a semi-reliable communication working mode (limited number of retransmissions). Nevertheless, distinct from the first, the node energy status is taken into account, such that, when the nodes have high energy level (requiring no energy savings), the network tends to operate in a working mode close to the full-reliable communication (limited but large number of retransmissions allowed). Then, gradually, the nodes switch to a saving resources working mode, such that all the network nodes converge to a single and finite number of retransmissions by packet (status similar to the first heuristic). Observe that, regardless the adopted heuristic, by using the proposed energy saving protocol the number of retransmissions per packet in the Hop-by-Hop scheme is always limited and it is possible to achieve substantially better energy consumption indexes in the communication link than using full-reliable network (compatible with classical control theory). To quantify the energy savings of the proposed protocol, it is used a stochastic model for the multi-hop network, which provides average expected energy consumption for the entire network. Another important parameter that is investigated is the energy consumed by each individual node, because it allows to determine the node with lowest path lifetime and also identify the network nodes that are more susceptible to failure due to energy consumption issues.

The proposed resource saving protocol is formulated in a generalist structure, that is, it can be applied to any NCS problem with multiple information sources, such as filtering, state-feedback, static output-feedback or dynamic output-feedback control. Nevertheless, to show the viability of employment and main features of the protocol, the problem of output-feedback control via WSN is particularly investigated in this paper. To deal with the packet loss in the communication channel, the controlled plant is modeled as a discrete-time Markov Jump Linear System (MJLS), whose jumps between the operation modes can represent the possible transmission failures [16,17]. The MJLS class allows to model the success or failure transmission simply changing the values of the input and output matrices of the system [18]. As previously mentioned, the performance criterion adopted to synthesize the controler is the $H_{\infty }$ norm of the closed-loop system [19].

Another important contribution of this paper is the proposition of a systematic procedure to determine the transition probability matrix and the modeling of the Markov chain operation modes. Since several measurement signals can be read by a single sensor (or control signals can be sent to the same actuator), the operation modes are determined by fail or success in the transmission of packets associated to the total amount of those devices (product between the number of sensors and actuators). Additionally, from the viewpoint of the remote controller, only the packet loss of the measurement signals can be recognized, indicating that some of the modes (associated with the control input) are not observable. To the best of the authors’ knowledge, there are only sub-optimal conditions for the design of dynamic output-feedback controllers in the partially mode-dependent context. On the other hand, concerning the design of filters (full-order or state-observer) and state-feedback controllers, there exist necessary and sufficient conditions even for the scenario regarding partial availability of the chain modes in the particular case of MJLS with generalized Bernoulli distribution [20,21], a problem investigated in this paper. For this reason, it was chosen to perform the design of the controller in two steps: synthesis of a state-observer filter and synthesis of a state-feedback gain. Although each part of the design is optimal, the joint design (filter + state-feedback controller) lost its optimality because the separation principle is not valid for $H_{\infty }$ control of MJLS. Even though, if all the system states are accessible for feedback or if the packet loss does not occur in the transmission of the measurement or control signals, the same design conditions provide the optimal performance.

The remainder of this paper is briefly summarized in what follows. Section 2 presents different strategies available in the literature to model the packet loss in WSN scenario. Section 3 introduces the mathematical background necessary for the presentation of the results proposed in the paper, such as the definition of the Markov model for dynamical systems controlled through communication network; the methods to design the $H_{\infty }$ output-feedback controller; the formulas for the calculation of the transmission success probability and mathematical expectation of the global number of transmissions using the Hop-by-Hop transport scheme; and the energy consumption model for WSN. Section 4 presents the modeling of the Markov operation modes and the computation of the probabilities of transition among them for problems of control through semi-reliable communication networks with multiple information sources. Section 5 describes in details the three heuristics of implementation of the proposed energy saving protocol. In Section 6 the energy saving protocol is applied to a standard control problem: the coupled water tank, a multiple-input multiple-output (MIMO) system with two inputs and two outputs. The numerical results in terms of the $H_{\infty }$ performance degradation and the reduction of the global energy consumption when compared with a classical control system (implemented in a full-reliable network) are also presented. Section 7 provides a critical assessment of the results and presents final considerations and some future perspectives. Appendix A presents a tutorial for the use of the R-Package ‘Hopbyhop’, which facilitates the computation of the transmission success probabilities and the mathematical expectation of the global number of transmissions in a Hop-by-Hop network and also provides a validation of the calculations by means of a Monte Carlo simulation.

## 2. General Background

Efficient energy consumption is a major concern in the WSN field and the specialized literature reveals a rich variety of manners for increasing the lifetime of a sensor network. Clearly, the adoption of different materials or hardware components may allow significant energy savings. In [22], Polastre et al. show how the choice of one kind of node over another can more than double the lifetime of a simple sensor application. Actually, different hardware will bring different consumptions for the very same applications, but the way in which those components are used (the logical part) is also relevant [23]. At the source level, energy-efficient data processing and storage algorithms must be adopted [24,25,26,27], while, at the network level, communication protocols at different network layers work for transporting the information between the source and the sink nodes through (potentially) several different intermediate nodes, in the most efficient manner (considering nodes restrictions and the application nature) [28,29,30]. Besides, note that even different deployment choices will impact the operation of the WSN [31], and, as a consequence, the consumption of the batteries.

This work investigates the problem of energy-aware WSN over NCS. Generally speaking, NCS are related to the use of communication channels shared by many interconnected units (so a network), with the purpose of closing one or several control loops [3]. This concept can be applied in a wide range of applications, such as in factory automation, tele-operation of robotic systems, health-care, smart cities, etc. The case of wireless NCS was already investigated in [32], identifying some associated problems such as packet loss and delay. The authors present a timing scheme and an adaptive control loop to cope with variations in network conditions. The reference wireless standard was IEEE 802.11b, which is designed to bring connectivity in Wireless Local Area Networks (WLAN) at data rates up to 11 Mbps. This standard is still used in laptops, cellphones and other mobile devices. The current paper is focused on WSN technologies which, as already mentioned, are most based on the IEEE 802.15.4 standard, focusing on low-rate data communications with low power consumptions. WSN-based NCS have been studied in many works. For instance, in [12], the authors propose a networked control system considering WSN issues. The controller is event-driven (event is a packet arrival) and includes a state predictor based on classical Extended Kalman Filter (EKF) to deal with delay. All the references mentioned in this paragraph consider WSN as responsible for producing data loss and delay, but the complexity of the network protocols and, particularly, the multi-hop case, are not considered. The investigation proposed in this paper treats various problems found in WSNs, including multi-hop transport of data and energy consumption. Even when not explicit in mathematical models, the packet loss is discussed in all related works as an inherent and relevant problem of WSN-based NCS. Among the different ways of considering packet loss, the most common ones can be classified as:

i **Unreliable protocols and robust codification.** In many cases, real-time requirements or resource limitations may force the use of unreliable protocols, i.e., protocols that do not assure the correct reception of all transmitted packets. In this case, the receiving application must be able to work with partial quantities of data. In many WSN-based applications, reliability is not a requirement because there exists a high correlation between the gathered data over time (or space). For instance, in weather monitoring applications, where sensors nodes send measures that do not present fast variations over time (thus losses can be easily estimated with the correctly received data). Another example is multimedia communication, where high correlations between a measure (e.g., a pixel on an image, or a level of sound) and the surrounding ones exist (on space or time). Additionally, in order to support the reconstruction process, robust codes at the sender side may be included. That is the case of the interleaving techniques studied in [33].
ii **ARQ-based full-reliable protocols.** In situations where the reception of the complete set of transmitted packets must be ensured, Automatic Repeat reQuest (ARQ) protocols can be adopted. ARQ follows these simple steps: (1) after receiving a packet, the receiver sends back to the sender an acknowledgment packet (ACK); (2) the sender waits, for a certain period of time, for the ACK signal and (3) if the corresponding ACK is not received, the sender retransmits the data packet. Many state-of-the-art protocols and standards use ARQ as a basis, including Transmission Control Protocol(TCP), Carrier sense multiple access with collision avoidance Media Access Control (CSMA/CA MAC) protocols [34].
iii **FEC and redundancy.** For enhancing reliability of a communication system, Forward Error Correction (FEC) or redundancy techniques may be adopted. FEC is applied in unreliable channels (with no ARQ protocol), and relies on adding redundant bits for allowing the decoder to retrieve the original data. Redundant packets can also be transmitted to avoid packet loss between source and sink, because the chance of a given data, among several copies, to reach the decoder increases with the number of copies sent.
iv **Semi-reliable protocols.** In WSN, semi-reliable communication of prioritized data packets can be adopted to achieve some energy savings requirements. Some transforms (e.g., Wavelet, etc.) may allow to separate different components of an input signal. Generally, the transmission of a small set of components is enough to retrieve the original signal. Thus, the components are transmitted with different levels of reliability, according to their importance on the reconstruction of the final signal and other decision information (which is called a semi-reliable protocol). In [35,36], this concept is adopted in a communication protocol specialized in transmitting data associated to pictures in a semi-reliable manner, aiming to save energy resources. In both works, the original image is split in different coefficient levels by a 2-Dimensional Discretized Wavelet Transform (2D-DWT). The resulting coefficients are grouped by wavelet level, with decreasing priorities starting with the approximated image by a low-pass filter (the highest priority). Then, a semi-reliable protocol is executed by the intermediate nodes, which are able to either (reliably) forward or not an incoming packet considering its associated priority level and the corresponding batteries state-of-charge of the nodes. This protocol was extended in [37], allowing nodes to decide the retransmission of packets based not only on their own batteries status, but also on the following nodes on the path towards the sink. This particular mechanism has inspired the proposal of this paper, as commented in Section 4 and Section 5.

## 3. Preliminaries

### 3.1. Notation

The following notation is used in this manuscript:


N
Set of natural numbers
$R^{n}$
*n*th dimensional Euclidean space with the
usual norm ∥·∥
$\left(\Omega ,F,\left\{F_{k}\right\},\Gamma \right)$
Fundamental probability space
σ
number of Markov operation modes
$K=\left\{1,\cdots ,\sigma \right\}$
finite set with all Markov operation modes
$\theta_{k}$
random variable that assume values on set K
$p_{ij}$
probability of transition from mode θ(k)=i to θ(k+1)=j
$P^{\sigma \times \sigma }=\left[p_{ij}\right]$
Transition probability matrix
$L_{2}$
class of stochastic signals $\zeta \left(k\right)\in R^{r}$, ∀k∈N, such that ${∥\zeta ∥}_{2}^{2}=\sum_{k=0}^{\infty }E\left\{\zeta {\left(k\right)}^{\prime }\zeta \left(k\right)\right\}$
is finite
E{·}
mathematical expectation
${\left(\cdot \right)}^{\prime }$
transposition
${\left(\cdot \right)}^{-1}$
inverse(•)block induced by symmetry
$E_{TX}$
transmission mode energy consumption
$E_{RX}$
reception mode energy consumption
$E_{SW}$
energy spent during the switching between TX and RX
$E(E_{T}\left(p,L,N\right))$
energy consumed by a network responsible for the transport of the control signal
$L_{p_{TX}}$
packet size transmitted
$L_{p_{RX}}$
packet size received
$P_{S}$
success rate of transmission of a packet from the source to the sink
E(M)
expected number of global transmissions in the network
*p*
probability of transmission success per packet among nodes
*N*
number of communication links
*L*
maximum number of allowed transmissions per packet
$T_{S}$
sampling time
Ξ
dimensionless value used to adjust the amount of time⊗Kronecker product
$N_{U}^{R,S}$
indicates that node U belongs to the routes of set *R* transmitting signals of set *S*
$T_{R}$
set of all routes
$T_{S}$
set of all packets of signals
Ψ
set of all nodes belonging to the network
$N_{max}\left(N_{U}^{R,S}\right)$
node with highest energy consumption (highest value of E(M) per hop)
$E_{GE}$
global energy consumption (quantified in J/hour)
ξ
network setting index
$I_{L_{m}}$
sequence of the network setting

### 3.2. Markovian Model

Consider the following discrete-time MJLS G, on the probabilistic space $\left(\Omega ,F,\{F_{k}\},\Gamma \right)$, described by a set of difference equations given by:

(1) $$G≜\left\{\begin{array}{cc} x(k+1) & =A\left(\theta_{k}\right)x\left(k\right)+B\left(\theta_{k}\right)u\left(k\right)+E\left(\theta_{k}\right)w\left(k\right)\\ y(k) & =C_{y}\left(\theta_{k}\right)x\left(k\right)+E_{y}\left(\theta \right)w\left(k\right),\\ z(k) & =C_{z}\left(\theta_{k}\right)x\left(k\right)+D_{z}\left(\theta_{k}\right)u\left(k\right)+E_{z}\left(\theta_{k}\right)w\left(k\right), \end{array}\right.$$

where $x\left(k\right)\in R^{n_{x}}$ is the state vector, $u\left(k\right)\in R^{n_{u}}$ is the control input and $w\left(k\right)\in R^{n_{w}}$ is the noisy input. Vector $y\left(k\right)\in R^{n_{y}}$ is the measurement output and $z\left(k\right)\in R^{n_{z}}$ is the controlled output. To ease the notation, whenever $\theta_{k}=i$, one writes $A\left(\theta_{k}\right)=A_{i}$, $B\left(\theta_{k}\right)=B_{i}$ and so forth, ∀i∈K.

To design $H_{\infty }$ stabilizing controllers for system (1), firstly it is necessary to present the concept of stability for MJLS. This definition, named as *mean square stability* (MSS) [38], ensures that $E\left[∥x(k)∥\right]\to 0$ as k→∞ for any initial condition $x_{0}\in R^{n_{x}},\;\theta_{0}\in K$.

#### 3.2.1. $H_{\infty }$ Norm Computation for MJLS

The $H_{\infty }$ norm for MJLS is defined as a ratio between the expected values of the exogenous input w(k) and the output z(k) for the worst case scenario of the signal $w\left(k\right)\in L_{2}$. One of the possible definitions of the $H_{\infty }$ norm of an MJLS is presented in [39], and reproduced below,

(2) $${∥G∥}_{\infty }^{2}=\underset{0\neq w\in L^{2},\;\theta_{0}\in K}{\sup}\frac{{∥z\left(k\right)∥}_{2}^{2}}{{∥w\left(k\right)∥}_{2}^{2}}.$$

The $H_{\infty }$ norm, that is finite only if the MJLS (1) is MSS, can be calculated by solving a convex optimization problem in the form of Linear Matrix Inequalities (LMIs) [40,41] as presented next.

**Lemma** **1.** *MJLS* (1) *in open-loop (u(k)=0) is MSS and satisfies ${∥G∥}_{\infty }<\gamma$ if and only if there exist symmetric positive definite matrices $P_{i}$, such that the LMIs*

(3) $${\left[\begin{array}{cc} A_{i} & E_{i}\\ {C_{z}}_{i} & {E_{z}}_{i} \end{array}\right]}^{\prime }\left[\begin{array}{cc} P_{pi} & 0\\ 0 & I \end{array}\right]\left[\begin{array}{cc} A_{i} & E_{i}\\ {C_{z}}_{i} & {E_{z}}_{i} \end{array}\right]-\left[\begin{array}{cc} P_{i} & 0\\ 0 & \gamma^{2}I \end{array}\right]<0$$

*hold for each i∈K with $P_{pi}=\sum_{j=1}^{\sigma }p_{ij}P_{j}$.*

#### 3.2.2. Full Order Dynamic Output-Feedback Controller for MJLS

A full-order dynamic output-feedback controller for system (1) with the best (optimal) $H_{\infty }$ performance can be obtained with the design method given in [42] if all Markov operation modes (i∈K) are accessible. Since this assumption is unrealistic in many applications, leading to only suboptimal solutions when employing the technique of [42], in this paper a different output-feedback approach is pursued, based on the separated design of a state-observer filter and a state-feedback controller. Although in the WSN-based NCS literature there are plenty of works dealing with the design and implementation of filters and controllers, the proposed approach is described in the MJLS framework associated with a Markov chain with Bernoulli distribution. In this particular case, even when the operation modes are not available, both the obtained filter [20] and controller [21] provide optimal solutions individually.

Concerning the design of the state-observer, consider a Luenberger filter F (internal model-based filter), given by

(4) $$F:\left\{\begin{array}{cc} x_{f}\left(k+1\right) & =A_{i}x_{f}\left(k\right)\;+{G_{f}}_{i}\left(y\left(k\right)-C_{yi}x_{f}\left(k\right)\right),\\ z_{f}\left(k\right) & =C_{zi}x_{f}\left(k\right)+{K_{f}}_{i}\left(y\left(k\right)-C_{yi}x_{f}\left(k\right)\right), \end{array}\right.$$

where $x_{f}\left(k\right)\in R^{n_{x}}$, $z_{f}\left(k\right)\in R^{n_{z}}$ and $y\left(k\right)\in R^{n_{y}}$, are, respectively, the filter state vector, the estimated output and the measured signal output. Additionally, the filter matrices are given by ${A_{f}}_{i}=\left\{A_{i}-{G_{f}}_{i}C_{yi}\right\}$, $B_{f}={G_{f}}_{i}$, ${C_{f}}_{i}=\left\{C_{zi}-{K_{f}}_{i}E_{yi}\right\}$ and $D_{fi}=K_{f}$, where ${G_{f}}_{i}$ and ${K_{f}}_{i}$ are the matrices to be designed. The estimation error is defined as $e\left(k\right)=z\left(k\right)-z_{f}\left(k\right)$, and its dynamics is represented by the set of equations given in (5), connecting the filter (4) to the MJLS (1) with u(k)=0

(5) $$G_{o}:\left\{\begin{array}{cc} \tilde{x}\left(k+1\right) & =\left(A_{i}-{G_{f}}_{i}C_{yi}\right)\tilde{x}\left(k\right)+\left(E_{i}-{G_{f}}_{i}E_{yi}\right)w\left(k\right),\\ e(k) & =\left(C_{zi}-{K_{f}}_{i}C_{yi}\right)\tilde{x}\left(k\right)+\left(E_{zi}-{K_{f}}_{i}E_{yi}\right)w\left(k\right), \end{array}\right.$$

where $\tilde{x}\left(k\right)=x\left(k\right)-x_{f}\left(k\right)$. As mentioned before, the assumption of complete Markov mode availability is not realistic. Thus, in some applications, it may be more adequate to consider cluster availability. Therefore, consider the set $L=1,2,\ldots ,\sigma_{c}$ with $\sigma_{c}\leq \sigma$ and define the set of Markov chain states K as the union of $\sigma_{c}$ disjoint sets, or clusters, that is, 
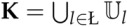
 such that $U_{i}\cap U_{j}=\emptyset$, ∀i=j∈L. The matrices of filter (4) designed in the case $z\left(k\right)=\hat{x}\left(k\right)$ (state-observer filter) can be synthesized by solving a set of LMI conditions presented in [21] and reproduced in the sequence for convenience.

**Lemma** **2.** *There exists a filter as in Equation* (4), *such that $∥G_{o}{∥}_{\infty }^{2}<\gamma$, under the assumption of $p_{ij}\;=\;p_{j}\;\forall i,j\in K$, if and only if there exist symmetric matrices $H_{i}$, X, and matrices $F_{l}$, $K_{l}$ with compatible dimensions fulfilling the LMIs*

(6) $$\left[\begin{array}{cccc} H_{i} & • & • & •\\ 0 & \gamma I & • & •\\ XA_{i}+F_{l}C_{yi} & XE_{i}+F_{l}E_{yi} & X & •\\ C_{zi}-K_{l}C_{yi} & E_{zi}-K_{l}E_{yi} & 0 & I \end{array}\right]>0,\forall i\in K,\forall l\in L,$$

*and*

(7) $$H_{p}-X<0,$$

*where $H_{p}=\sum_{j=1}^{\sigma }p_{j}H_{j}$. If a feasible solution is achieved, the filter gains are given by*

(8) $${G_{f}}_{l}=-X^{-1}F_{l}\;\;\;\;and\;\;\;\;{K_{f}}_{l}=K_{l}\;\;\forall l\in L.$$

On the other hand, consider the general problem of designing a state-feedback control law

(9) $$\begin{array}{c} u\left(k\right)=K_{i}x\left(k\right), \end{array}$$

for system (1) where $K_{i}$ are state-feedback gains to be designed. Considering y(k)=x(k) in Equation (1), the closed-loop system is given by

(10) $$G_{k}:\left\{\begin{array}{cc} \hat{x}\left(k+1\right) & =\left(A_{i}+B_{i}K_{i}\right)x\left(k\right)+E_{i}w\left(k\right),\\ z(k) & =\left({C_{z}}_{i}+D_{z_{i}}K_{i}\right)x\left(k\right)+{E_{z}}_{i}w\left(k\right) \end{array}\right.$$

Considering a generalized Bernoulli distribution for MJLS (1), the state-feedback control law (9) that assures an upper bound for the $H_{\infty }$ norm and the MSS of the closed-loop MJLS (10) can be obtained by solving the LMI conditions given in [20], reproduced as follows.

**Lemma** **3.** *There exists a state-feedback controller* (9), *such that $∥G_{k}{∥}_{\infty }^{2}<\gamma$ if and only if there exist symmetric matrices $Z_{i}$ and X, and a matrix $Y_{l}$ with compatible dimensions satisfying the LMIs*

(11) $$\left[\begin{array}{cccc} Z_{i} & • & • & •\\ 0 & \gamma I & • & •\\ A_{i}X+B_{i}Y_{l} & E_{i} & X & •\\ C_{z_{i}}X+D_{zi}Y_{l} & E_{z_{i}} & 0 & I \end{array}\right]>0,\forall i\in K,\forall l\in L,$$

(12) $$Z_{p}-X<0,$$

*where $Z_{p}=\sum_{j=1}^{\sigma }p_{j}Z_{j}$. If a feasible solution is found, the partially mode-dependent state-feedback control gains are given by $K_{l}=Y_{l}X^{-1}$.*

System (1) associated with the filter-observer ($z_{f}\left(k\right)=x\left(k\right)$) given in Equation (4) and the state-feedback control law (9) with $x\left(k\right)=z_{f}\left(k\right)$ can be rewritten as

(13) $$G:\left\{\begin{array}{cc} \hat{x}\left(k+1\right) & ={\hat{A_{o}}}_{i}\hat{x}\left(k\right)+{\hat{B_{o}}}_{i}w\left(k\right)\\ z(k) & ={\hat{C_{o}}}_{i}\hat{x}\left(k\right)+{\hat{D_{o}}}_{i}w\left(k\right) \end{array},\right.$$

with $\hat{x}={\left[x{\left(k\right)}^{\prime }\;x_{f}{\left(k\right)}^{\prime }\right]}^{\prime }\in R^{2n_{x}}$, and the following matrices

(14) $$\begin{array}{c} {\hat{A_{o}}}_{i}=\left[\begin{array}{cc} A_{i}+B_{i}K_{i}D_{fi}C_{yi} & B_{i}K_{i}C_{fi}\\ B_{fi}C_{yi} & A_{fi} \end{array}\right],\;{\hat{B_{o}}}_{i}=\left[\begin{array}{c} E_{i}+B_{i}K_{i}D_{fi}E_{yi}\\ B_{fi}E_{yi} \end{array}\right],\\ {\hat{C_{o}}}_{i}=\left[\begin{array}{cc} C_{zi}+D_{zi}K_{i}D_{fi}C_{yi} & D_{zi}K_{i}C_{fi} \end{array}\right],\;{\hat{D_{o}}}_{i}=\left[\begin{array}{c} E_{zi}+D_{zi}K_{i}D_{fi}E_{yi} \end{array}\right]. \end{array}$$

When the matrices of system (13) are known, the $H_{\infty }$ norm can be computed using the conditions of Lemma 1. However, it is important to emphasize that Lemmas 2 and 3 provide optimal solutions for partially mode-dependent filter and static state-feedback controller when considering a generalized Bernoulli distribution (transition probability matrix with identical rows) for each problem solved independently. When the solutions are combined, only a suboptimal solution in terms of the $H_{\infty }$ performance is obtained. Actually, the mode-independent output-feedback is an open problem in the MJLS literature (only suboptimal solutions are available so far).

### 3.3. Fundamental Concept of the Energy-Efficiency

Consider a network composed by a set of communication units (nodes). Each unit usually has two operation modes: transmission mode TX and reception mode RX. Each operation mode has a specific energy cost. In addition, the commutation between TX and RX also has a energetic cost associated. The costs are defined as: $E_{TX}$ (denoting the transmission mode energy consumption), $E_{RX}$ (denoting the reception mode energy consumption), and $E_{SW}$ (denoting the energy spent during the switching between TX and RX). The transmission and reception energy costs depend on the packet size being transmitted, $Lp_{TX}$ and $Lp_{RX}$.

The energy consumption associated with a transmission of a single packet is explicitly dependent on the hardware and the packet size (in bytes) [37], however, in a multi-hop network the total energy cost depends not only on the hardware but also on the reliability of the protocol implemented in the network. These protocols are responsible to set the packet retransmissions policy, and they are commonly used to ensure that the packets certainly arrive, drastically increasing the network reliability. To quantify the energy cost, the models used in this work are presented in [37], but models associated to other types of technologies could be used.

#### 3.3.1. Energy Consumption for Hop-by-Hop Networks

For the multi-hop network where the Hop-by-Hop transport scheme is implemented, it is possible to model the transmission failure and success using a Bernoulli stochastic process, where $P_{S}$ and E(M), that respectively represent the success rate and the expected number of global transmissions in the network, can be theoretically obtained by using the following lemma.

**Lemma** **4.** *For a system in which a transport scheme Hop-by-Hop is implemented, the packet arrival success probability is given by:*

(15) $$P_{S}={\left[1-{\left(1-p\right)}^{L}\right]}^{N}$$

*and the mathematical expectation of the global number of transmissions depends on the variables L, N and p, given by:*

(16) $$E\left(M\right)=\left\{\begin{array}{cc} \frac{\left[1-{\left(1-p^{2}\right)}^{L}\right]}{p^{2}{\left(1+p\right)}^{-1}}\left[\frac{1-{\left[1-{\left(1-p\right)}^{L}\right]}^{N}}{{\left(1-p\right)}^{L}},\right] & if\;L<\infty ,\\ \frac{N(1+p)}{p^{2}}, & if\;L\;\mathrm{unlimited}. \end{array}\right.$$

*where p, N and L represent, respectively, the probability of transmission success, the number of communication links and maximum number of transmissions per packet.*

Equations (15) and (16) impose that the probability of success of a data packet transmission and the probability of success of the acknowledgment packets must have the same value. The proof for Equation (16) in Lemma 4 appears in [6], such that it can be calculated using a computational package [43] (*HBH ($p_{1}$, $p_{2}$, L, N)* for R Package ‘hopbyhop’ with $p=p_{1}=p_{2}$).

#### 3.3.2. Energy Consumption Model for WSN

Based on the cost values presented in [9] and [13] Chapters 3 and 7, of a multi-Hop network with the transport scheme Hop-by-Hop, according to Lemma 4, the energy consumed by a network responsible for the transport of the control signal is given by

(17) $$E\left(E_{T}\left(p,L,N\right)\right)\;=\Xi \times \frac{\left(E_{TX}+2E_{SW}+pE_{RX}\right)E\left(M\right)}{T_{S}}$$

where $T_{S}$, called *sampling time*, is the time interval used to transmit measured and control signals in a digital control system. The value of Ξ is dimensionless, and it is used to adjust the amount of time. The proof of Equation (17) is fully discussed in [9,13] for Bernoulli loss processes.

## 4. Modeling of the Markov Modes and Transition Probability Matrix for a Network with Multiple Information Sources

This section provides the definition of a control structure through WSN based on the client-server control architecture. Additionally, this section presents the modeling of the transition probability matrix and the operation modes for output-feedback control design in semi-reliable communication networks with multiple sources of information.

### 4.1. Client-Server Control Architecture

**Figure 1:**
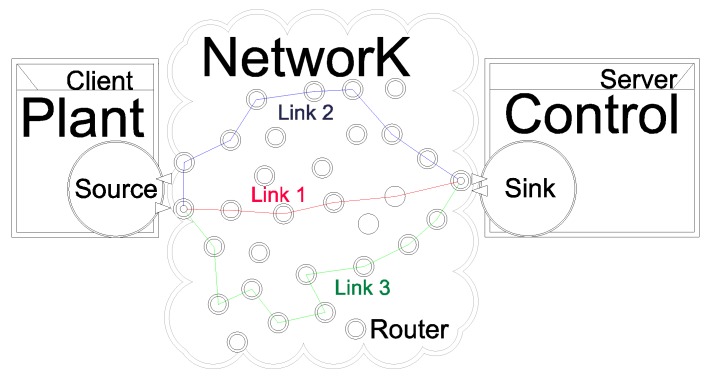
Cliente-Server for NCS.

A control design scheme where the connection among the elements is made through digital networks can be summarized as a Client-Server control architecture [3], as depicted in Figure 1. The client is the controller (Algorithm), the server is the plant (Dynamic System), and the control loop is denoted as a multi-hop network that, in the case investigated in this paper, is represented by a Wireless Sensor Network.

The packets are transported via a topology chain using hops between intermediary terminals (router nodes) from the Source to the Sink.

The classical control schemes are designed assuming that the communication between all the elements is made via an ideal channel, meaning that the packet loss rate must be null. However, in real network applications, more specifically in a WSN (presented in Figure 1), to perform a network communication without packet loss is a troublesome task. Thus, to reach such ideal conditions, some packet flow control mechanisms are implemented to get as close as possible a full-reliable communication. These mechanisms usually perform re-transmissions and use acknowledgment signals. As examples, one can mention the transport layer End-to-End and Hop-by-Hop schemes [44]. To accomplish such level of reliability in a WSN generally it is required a high energy cost, because each unit must retransmit the packet by an unknown number of times until the packet arrives to the proper destination and the acknowledge signal (ACK) is properly received by the sink.

### 4.2. Operation Modes Associated with Output-Feedback Control of A Dynamical System through A Semi-Reliable Network

Using the client-server scheme shown in Figure 1, it is proposed a standard formulation to devise a transition probability matrix of the Markov model, supposing the existence of multiple sensors and actuators. In this scheme, the source (in this case, the set of sensors) sends a set of measurements (elements composing vector y(k)) to the sink (in this case, the controller). Subsequently, the controller (now acting as the source) sends the control signal u(k), which is received by the plant (sink). In the classical control, the number of independent sensors that generate the signal (y(k)) and the number of actuators that receive the signal (u(k)) are irrelevant due to the hypotheses that they are accessible in every time instant *k*. When considering the packet loss, one can model the measurement and control vectors as a composition of some subsets extracted from the original vectors if the signals composing those vectors are emitted by a set of different sources.

The classical control case assumes that all elements composing the vectors of measurement and control signals are correctly transmitted. However, in a plant subject to communication failure, there are σ operation modes representing different combinations of the original vectors, associated with the success and failure in the packet transmission, that is, the total number of modes can be obtained using

(18) $$\sigma =\sigma_{y}\sigma_{u}=2^{n_{\bar{y}}+n_{\bar{u}}},\;\;n_{\bar{y}}\leq n_{y},\;\;n_{\bar{u}}\leq n_{u},$$

since $\sigma_{y}=2^{n_{\bar{y}}}$ and $\sigma_{u}=2^{n_{\bar{u}}}$, where $n_{\bar{y}}$ and $n_{\bar{u}}$ represent the number of independent sensors and independent actuators, such that those values can not surpass the size of vectors y(k) ($n_{y}$) and u(k) ($n_{u}$). Such limitation is motivated by the fact that a single sensor (or actuator) may send (or receive) a packet with different types of information. As an example, one can mention an encoder that provides both the velocity and the position at the same time. Furthermore $n_{\bar{y}}$ and $n_{\bar{u}}$ also denote the number of elements of the vectors of packets of measurement signals ($\bar{y}=\left[{\bar{y}}_{1},\ldots ,{\bar{y}}_{n_{\bar{y}}}\right]$) and control input signals ($\bar{u}=\left[{\bar{u}}_{1},\ldots ,{\bar{u}}_{n_{\bar{u}}}\right]$).

#### 4.2.1. Success Probability and Chain Mode Accessibility

The total number of operation modes σ of system (1) also corresponds to the order of the Markov chain, such that $P\in R^{\sigma \times \sigma }$ models the probability of transition between the modes. In this paper two sets of particular events are defined: *i)* The success and failure of transmission of elements of y(k), which is modeled by $P^{y}\;\in R^{\sigma_{y}\times \sigma_{y}}$ whose set of operation modes is given by $\ell_{y}\in K^{y}=\left[1,\ldots ,\sigma_{y}\right]\in K$; and *ii)* the success and failure of transmission of the elements of u(k), which is modeled by $P^{u}\;\in R^{\sigma_{u}\times \sigma_{u}}$ such that each operation mode is represented by $\ell_{u}\in K^{u}=\left[1,\ldots ,\sigma_{u}\right]\in K$. The general process that comprises the entire system is associated with the Kronecker product of the two transition probability matrices, that is, $P^{u}⊗P^{y}=P\in R^{\sigma \times \sigma }$, that governs the jumps between the modes of the global Markov process.

Each element in the transition matrix $P=[p_{ij}]$ represents the probability of transition of the current state *i* to the state *j*. This transition may be used as a model for the network communication scheme. In the present work, a Multi-Hop network topology that implements the Hop-by-Hop transmission scheme is used. The stochastic process that determines the success or failure of transmission uses the generalized Bernoulli distribution model, which means $p_{ij}=p_{j}$, so the probability does not depend on current state (index *i*).

For illustrative purposes, consider that system (1) has a vector of output measurements with 3 components ($y\left(k\right)\in R^{3}$) but only two sensors are connected to the plant ($n_{\bar{y}}=2$), such that the first two components of y(k) are simultaneously provided by the first sensor and the last component is given by the second sensor (${\bar{y}}_{1}=\left\{y_{1}\left(k\right),y_{2}\left(k\right)\right\}$ e ${\bar{y}}_{2}=\left\{y_{3}\left(k\right)\right\}$). Neglecting the packet loss of the control signal, considering only the transmission of the measurement output, one has $\sigma_{y}=2^{n_{\bar{y}}}=2^{2}=4$ operation modes and the set of the operation modes associated with y(k) is $\ell_{y}\in K^{y}=\left[1,\ldots ,\sigma_{y}\right]=\left[1,2,3,4\right]$. Knowing that the process of transition among the modes $\ell_{y}$ follows a generalized Bernoulli distribution (uncorrelated events), such that it does not depend on the initial state but only on the final state ($p_{ij}=p_{j}$), from the formula proposed in Lemma 4 one can compute the probability of occurrence of each event (success or failure in the transmission of ${\bar{y}}_{i}$, i=1,2):

(19) $$P_{{\bar{y}}_{i}}=\mathrm{Pr}\left({\bar{y}}_{i}\right)={\left[1-{\left(1-p\left({\bar{y}}_{i}\right)\right)}^{L({\bar{y}}_{i})}\right]}^{N({\bar{y}}_{i})}.$$

The possible events associated to the success or failure in the transmission of the measurement output is shown in Table 1.

Additionally, suppose that system (1) has one vector of control inputs with 4 components ($u\left(k\right)\in R^{4}$) and only two actuators ($n_{\bar{u}}=2$), such that the first two components of u(k) are implemented in the same actuator (${\bar{u}}_{1}=\left\{u_{1}\left(k\right),u_{2}\left(k\right)\right\}$) and the other two components are implemented in the second actuator (${\bar{u}}_{2}=\left\{u_{3}\left(k\right),u_{4}\left(k\right)\right\}$). The number of operation modes associated to the transmission of the control signal is $\sigma_{u}=2^{n_{\bar{u}}}=2^{2}=4$, such that $\ell_{u}\in K^{u}=\left[1,\ldots ,\sigma_{u}\right]=\left[1,2,3,4\right]$. Knowing that the process of transition among the modes $\ell_{u}$ also follows a generalized Bernoulli distribution, one can compute the probability of occurrence of each event $\ell_{u}=i$, $i=1,\ldots ,\sigma_{u}=1,\ldots ,4$, by:

(20) $$P_{{\bar{u}}_{i}}=\mathrm{Pr}\left({\bar{u}}_{i}\right)={\left[1-{\left(1-p\left({\bar{u}}_{i}\right)\right)}^{L({\bar{u}}_{i})}\right]}^{N({\bar{u}}_{i})}.$$

The possible events associated with success or failure of transmission of the control input are shown in Table 2.

Finally, the number of operation modes of the general process is $\sigma =\sigma_{y}\sigma_{u}=4\times 4=16$, representing all the possible combinations of the events associated with system (1), as described below:

Mode j∈K12345678910111213141516
$\ell_{y}\in K^{y}$
1111222233334444
$\ell_{u}\in K^{u}$
1234123412341234

Knowing that the transition probability matrix of the general process P is given by the Kronecker product of $P^{y}$ and $P^{u}$, the elements of $P=\left[p_{ij}\right]=\left[p_{j}\right]$ can be calculated by

$$p_{j}=\mathrm{Pr}\left(j\right)=\mathrm{Pr}\left(\ell_{y}\right)\times \mathrm{Pr}\left(\ell_{u}\right).$$

Particularly, for the mode j=10, one has

$$\mathrm{Pr}\left(j=10\right)=\mathrm{Pr}\left(\ell_{y}=3\right)\times \mathrm{Pr}\left(\ell_{u}=2\right)=\left(1-P_{{\bar{y}}_{1}}\right)P_{{\bar{y}}_{2}}\times P_{{\bar{u}}_{1}}\left(1-P_{{\bar{u}}_{2}}\right).$$

Notice that the operation mode is direct associated with both sets of events ($\ell_{y}\in K^{y}$ and $\ell_{u}\in K^{u}$). For this reason, if any of the operation modes associated separately with *u* or *y* is not accessible, it is not possible to know which packet was correctly transmitted and the only feasible option is to employ mode-independent controllers or filters. A recent approach presented in [45,46,47,48] proposes a synthesis procedure where the filters/controllers no longer depend on the access to the actual operation mode *i* of the Markov chain, instead, they depend on the estimated operation mode *l* of the Markov chain. This kind of approach seems appealing, however, the estimation algorithm in the implementation process increases the complexity of the solution proposed in this paper. Another solution, more widely exploited, is the use of clusters, meaning that the Markov chain modes are organized in subsets. In the case under investigation, the information about the events $\ell_{u}\in K^{u}$ is considered not accessible. On the other hand, the events $\ell_{y}\in K^{y}$ are completely observable, that is, it is always possible to know if a set of measurements (${\bar{y}}_{i}$, $i=1,\ldots ,\sigma_{y}$) was correctly transmitted. For this reason, this paper proposes the use of an approach based on *clusters*, that are mutually exclusive groups whose union generates the set of states K. In this sense, the operation modes of system (1) are grouped in $\sigma_{y}$ disjoint subsets, such that $\ell \in [1,\ldots ,\sigma_{y}]$. For the particular case of the illustrative example, instead of designing 16 controllers (associated with σ=16 distinct operation modes), only 4 controllers must be designed (associated with $\sigma_{y}=4$ distinct clusters), constituting a partially mode-dependent control technique. It is important to emphasize that dynamic output-feedback control synthesis conditions that are necessary and sufficient for the case of incomplete availability of the Markov states do not exist, meaning that only sub-optimal solutions are available in the literature for this scenario.

## 5. Energy Saving Protocol

Three distinct heuristics of the energy saving protocol are investigated in this paper.

- **Trade-off approach:** The first technique consists in the use of a semi-reliable network with a single and finite value for the maximum number of transmissions allowed (L<∞) in the global network. Consequently, due to the limitation of *L*, the probability of successful communication for each packet is less than one. Despite this disadvantage, the limitation of *L* provides energy-savings in the use of network resources. This protocol was proposed in [6,8,13].
- **Energy for node approach:** The second method is based on image transmission in WSN [15], where the probability of successful communication per packet between nodes is directly associated with the energy level of the transmission for a particular node. The energy level of the nodes starts at the maximum, which means that, initially, the probability of successful transmission of each node is equal to one (without packet loss, corresponding to the classical control theory, L=∞). After, according to the use of each node, its energy consumption increases, the value of *L* becomes finite and it is gradually reduced, implying a reduction in the successful transmission probability.
- **Mixed approach:** The third method is a mix between the previous approaches, that is, the probability of successful transmissions depends on the energy consumption of each network node, but the maximum number of transmissions allowed starts at a finite value. The idea is to obtain a performance as close as possible to classical control but maintaining desirable energy efficiency characteristics (L<∞). As a consequence, it is possible to achieve a better trade-off between performance and the use of network resources.

In order to perform a proper comparison between the classical control $P_{S}=1$ and the energy saving protocol proposed in this paper, some performance measurements variables are proposed:

- $Y_{H}=\frac{∥G_{cl}{∥}_{\infty }\left(L<\infty \right)}{∥G_{cl}{∥}_{\infty }\left(L\to \infty \right)}$ denotes the rate between the norm obtained with packet loss and the one using the classical approach (without loss).
- $Y_{M}=\frac{E[M|L<\infty ]}{E[M|L\to \infty ]}$ represents the ratio between the mathematical expectation of the global number of transmissions considering a limited value for *L* and the situation without any boundaries.
- $Y_{E}=\frac{E(E_{T}\left(p,L<\infty ,N\right))}{E(E_{T}\left(p,L=\infty ,N\right))}$ represents the ratio between the mathematical expectation of the global energy consumption using semi-reliable network and the energy consumption for full-reliable network (see (17)).
- $\Omega_{H}=Y_{H}-1$ is the normalized norm degradation arising from the use of a bounded *L*.
- $\Omega_{M}=1-Y_{M}$ is the normalized decrease of the mathematical expectation of the global number of transmissions due to the limitation of *L*.
- $\Omega_{E}=1-Y_{E}$ is the normalized decrease of the global energy consumption due to the limitation of *L*.
- $\Theta =\Omega_{M}-\Omega_{H}$ is the percentage difference between the normalized norm degradation and the normalized decrease of the mathematical expectation of the global number of transmissions due to the limitation of *L*.

The variable Θ was conceived with the task of verifying if there is any advantage in limiting the maximum number of transmissions per packet. Thus, if Θ>0, the decrease in the average number of global transmissions is proportionally greater than the increase in the $H_{\infty }$ norm, whereas Θ<0 represents the opposite. Therefore, when Θ>0, one assumes that the limitation of *L* is advantageous (positive trade-off), because it this implies a greater saving in energy resources than performance loss.

### 5.1. Network Nodes Nomenclature

A network may be understood as a collection of nodes interconnected through communication links (routes, see Figure 1). Each node can be part of different routes and can also transmit different packets (measurement and control signals) from the source to the sink. Additionally, the maximum number of transmissions *L* can be independent for each route and each node. To represent the network nodes in the energy saving protocols described in this section, the following indexes are used (by omitting the particular network structure)

$$N_{U}^{R,S}\in \Psi ,\;\;U=\left\{1,2,\ldots ,N_{node}\right\}$$

where *U* indicates which node is being evaluated, $N_{node}$ is the total number of nodes in the network, R and S respectively represent the number of routes and signals passing through *U* and Ψ denotes the set of all nodes belonging to the network. Furthermore, $T_{R}$ represents the set of all routes, such that $R\subset T_{R}$. Similarly, $T_{S}$ represents the set of all packets of signals, such that $S\subset T_{S}=K^{y}\cup K^{u}$. For example,

$$N_{10}^{\left[2,4\right],\left[{\bar{y}}_{2},{\bar{u}}_{1}\right]}$$

means that the node 10 belongs to the routes 2 and 4, transmitting the packets with signals ${\bar{y}}_{2}$ and ${\bar{u}}_{1}$.

### 5.2. Energy Saving Scheme by Trade-Off Approach

The use of the MJLS framework to represent a plant connected to the controller through a semi-reliable network enables the design of stabilizing controllers with theoretical guarantees of performance. By employing a similar Markovian model, it is also possible to design $H_{\infty }$ [6,7,8,13] and $H_{2}$ [10] filters connected through semi-reliable networks to the plant. However, related works [6,7,8,10,13] consider only the case of complete availability of the Markovian modes and that the measurement signals are provided by a single source of information. This means that the loss process could only be modeled by a Bernoulli transition probability matrix of order two, differently from the case handled in this paper, where the fragmentation of the signal vectors requires a transition probability matrix of higher order. Consequently, the partial mode-dependent approach is considered more adequate.

In the aforementioned works the decision variable used to optimize the use of network resources is the maximum number of transmissions per packet *L*. Thus, adapting the filtering approach for the control problem discussed in the Section 4, one has

$$\forall N_{U}^{R,S}\in \Psi \;\;\mathrm{using}\;\;L=\epsilon .$$

As discussed in Section 3.3.1, if ϵ is not limited, the success probability is given by $P_{S}=1$ and E(M) is the maximum possible value of the global number of transmissions, such that this setup corresponds to the classical control, without packet loss [6].

The trivial approach to save energy resources in a communication network is L=0, meaning that there is no transmission and, consequently, no energy consumption. However, not transmitting the control signal is not possible due to obvious reasons. In order to choose a proper value for *L*, the designer must define how much performance loss (in terms of the $H_{\infty }$ norm) is acceptable (when compared with the classical case: no packet loss) to achieve the energy savings that seems appropriate. A theoretical formulation of this problem is presented in the following lemma.

**Lemma** **5.** *For a given MJLS* (1) *associated to the transition probability matrix $P=\left[p_{ij}\right]=\left[p_{j}\right]$ computed according to the procedure presented in Section 4.2 with a finite L for some node $N_{U}^{R,S}$, if there exists a full-order dynamic output-feedback controller such that the closed-loop MJLS* (13) *satisfies Lemma 1, then this controller assures saving network resources when compared with the classical control design.*

### 5.3. Energy Saving Scheme by Energy for Node Approach

In WSN, the energy level of the autonomous units changes according to time (usually nonlinearly) and it can be estimated or measured by the battery voltage output [15]. When the battery life span is at the end, it is necessary to switch it to guarantee a good communication link, routes and control signal, otherwise the system may become unstable. Clearly, the best performance is attained by the classical digital controller, however, the energy consumption is maximum. Using the Trade-off approach (Lemma 5), the obtained $H_{\infty }$ performance is worse, but the energy consumption is improved.

Considering that initially all the network nodes have a good energy level, it would be convenient for the network to operate in a full-reliable working mode (no packet loss, L=∞), since this situation does not require energy savings. When a given node is near the end of its life span, it is appropriate to change its operation to energy savings working mode, extending its life span until a battery replacement. In this case, it is sufficient to limit the maximum number of retransmission (L=ϵ). However, besides the two working modes described above, it is possible to segment the battery life span in several working modes: starting from the full-reliable (L=∞) and going through a set of saving work modes, such that $L\in \{L_{1},\ldots ,L_{\kappa },\ldots ,L_{m}\}$ with $L_{1}=\infty$ and $L_{\kappa }\in N<\infty$, κ∈{1,2,…,m}, where *m* represents the total number of the network working modes.

**Figure 2:**
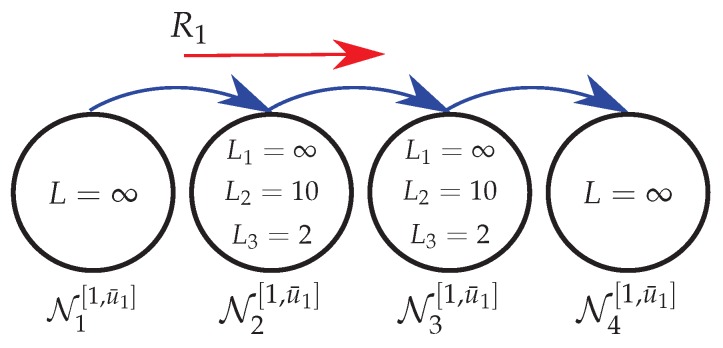
Illustrative network for the computation of P(ξ).

Since the switch of the working modes per node is associated with an external event (battery level), the nodes can work in different modes ($L_{\kappa }$), simultaneously. Each possible combination of $L_{\kappa }$ in the set of nodes Ψ corresponds to a different transition probability matrix P. The total number ξ of transition probability matrices is equal to the possible settings of the network, that is associated to the number of the working modes that each node has. For example, consider a network (illustrated in Figure 2) with 4 nodes (U={1,2,3,4}) with a single route R=1, transmitting a single signal packet $S=\{{\bar{u}}_{1}\left(k\right)\}$ such that the source and sink nodes ($N_{1}^{\left[1,{\bar{u}}_{1}\right]}$, $N_{4}^{\left[1,{\bar{u}}_{1}\right]}$) have unlimited energy, implying that the corresponding number of transmissions does not depend on the energy, i.e., $L_{1}=L_{4}=\infty$.

The nodes whose maximum number of transmissions *L* is limited correspond to $N_{2}^{\left[1,{\bar{u}}_{1}\right]}$ and $N_{3}^{\left[1,{\bar{u}}_{1}\right]}$, with three work modes (m=3) given by $L_{1}=\infty$, $L_{2}=10$ and $L_{3}=2$, generating ξ=9 network distinct settings:

$$\begin{array}{cc} \xi =1: & \Psi =\{N_{1}^{\left[1,{\bar{u}}_{1}\right]}\left(L=\infty \right),N_{2}^{\left[1,{\bar{u}}_{1}\right]}\left(L=L_{1}\right),N_{3}^{\left[1,{\bar{u}}_{1}\right]}\left(L=L_{1}\right),N_{4}^{\left[1,{\bar{u}}_{1}\right]}\left(L=\infty \right)\}\\ \xi =2: & \Psi =\{N_{1}^{\left[1,{\bar{u}}_{1}\right]}\left(L=\infty \right),N_{2}^{\left[1,{\bar{u}}_{1}\right]}\left(L=L_{1}\right),N_{3}^{\left[1,{\bar{u}}_{1}\right]}\left(L=L_{2}\right),N_{4}^{\left[1,{\bar{u}}_{1}\right]}\left(L=\infty \right)\}\\ \xi =3: & \Psi =\{N_{1}^{\left[1,{\bar{u}}_{1}\right]}\left(L=\infty \right),N_{2}^{\left[1,{\bar{u}}_{1}\right]}\left(L=L_{1}\right),N_{3}^{\left[1,{\bar{u}}_{1}\right]}\left(L=L_{3}\right),N_{4}^{\left[1,{\bar{u}}_{1}\right]}\left(L=\infty \right)\}\\ \xi =4: & \Psi =\{N_{1}^{\left[1,{\bar{u}}_{1}\right]}\left(L=\infty \right),N_{2}^{\left[1,{\bar{u}}_{1}\right]}\left(L=L_{2}\right),N_{3}^{\left[1,{\bar{u}}_{1}\right]}\left(L=L_{1}\right),N_{4}^{\left[1,{\bar{u}}_{1}\right]}\left(L=\infty \right)\}\\ \vdots \;\;\;\;\; & \vdots \\ \xi =9: & \Psi =\{N_{1}^{\left[1,{\bar{u}}_{1}\right]}\left(L=\infty \right),N_{2}^{\left[1,{\bar{u}}_{1}\right]}\left(L=L_{3}\right),N_{3}^{\left[1,{\bar{u}}_{1}\right]}\left(L=L_{3}\right),N_{4}^{\left[1,{\bar{u}}_{1}\right]}\left(L=\infty \right)\} \end{array}$$

Since each network setting is associated with a distinct transition probability matrix (P(ξ)) computed according to the procedures explained in Section 4.2.1, in this particular case, ξ=9 distinct sets of partially mode-dependent stabilizing controllers must be designed.

### 5.4. Energy Saving Scheme by Mixed Approach

The energy for node approach supposes that there exists the possibility of L=∞, that is, there is a time interval of full-reliability in the network and some other intermediate conditions to wait for a new battery. On the other hand, the trade-off approach assumes that L=ϵ is fixed and finite (lower than the classical case) for all the time. Joining both strategies, one has the *mixed approach* where *L* is always finite (semi-reliable network) but it can assume several distinct values (different network settings).

In this scenario, the communication network starts from an energy saving setup since the beginning. Therefore, the starting criterion ($L_{1}$) and the other working modes ($L_{\kappa }$, κ=2,…,m) must be chosen by analyzing the model adopted to represent the plant, such as the spectral radius (maximum absolute eigenvalue) of the dynamic matrix and the dynamics of the stochastic process associated to the packet loss model, taking into account that a wrong choice of $L_{\kappa }$ can lead the system to instability. Particularly, the maximum number of transmission allowed in the high energy level working mode ($L_{1}$) must be chosen by aiming at the best trade-off between the closed-loop performance and the energy consumption. A good choice is to set $L_{1}$ by following the lines presented in Åström and Wittenmark 1995: after performing a simulation of all communication network with a single value of $L_{\kappa }$, to compute its lower value such that Θ≥0 (that is, the percentage difference between the normalized performance degradation and the normalized decrease of the mathematical expectation by limiting *L* is not negative).

### 5.5. Validation Method

In [6], the Trade-off method (described in Section 5.2) was employed to optimize the network parameters. To evaluate the positive impact of this method in the communication channel, the only parameter used in [6] was the expected value of global transmissions in the network E(M), such that the lower this value the better the use of resources in the network. In a different way, this paper proposes the quantification of the positive impact on the network of the adopted energy saving scheme measured in terms of: (i) the expected value of energy consumed in all units that compose the control loop per unit of time, that can be computed using Lemma 4; (ii) the identification of the node whose expected value of energy consumed individually is the highest among the nodes ∈Ψ (computed by Appendix A borrowed from [43] along with Lemma 4). The first quantification criterion is useful for projections of, for instance, channel use and congestion. On the other hand, the node with the highest energy consumption is a critical one (quickest battery consume), because if its operation ends it can compromise the control loop. As example, in a semi-reliable network with a single route, the node closest to the source usually presents the highest energy consumption, while in a network with multiple-routes and a single sink, the node closest to the sink commonly is the critical one. Furthermore, the consumption of each node $N_{U}^{R,S}$ changes according with the total number of routes using this node, the type of transmitted signal, the probability of successful transmission and reception of those signals. The node with highest energy consumption is denoted by $N_{max}\left(N_{U}^{R,S}\right)\in \Psi$, corresponding to the node belonging to (An important practical consideration when determining the index *U* associated with the highest energy consumption node is that some units may be connected to other equipments, like sensors and actuators, that have their own energy consumption levels. Therefore, by excluding these special nodes with accumulated functions, only a subset of Ψ is considered) Ψ associated with the highest number of transmissions and receptions, that is, the highest value of E(M) per hop *N* (see Appendix A).

## 6. Example of Application of the Energy-Efficiency Protocol

In this section, an example is provided to illustrate the feasibility of the energy-efficiency protocol proposed in this paper. For a particular network, different energy saving schemes are tested in the control of a linearized model of a coupled multi-level tank.

### 6.1. Coupled Multi-Level Tank

**Figure 3:**
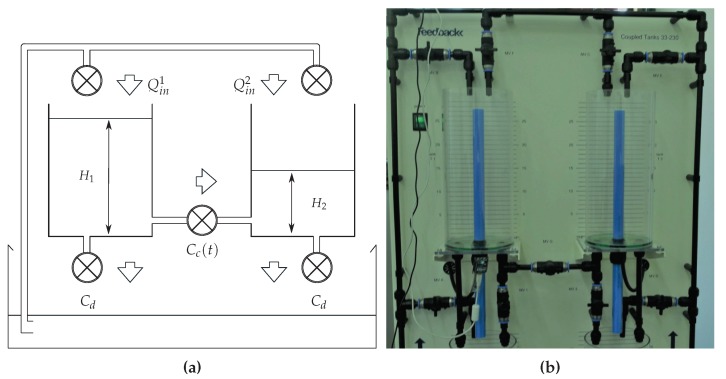
(**a**) Operating system diagram; (**b**) Level control plant used in the experiment.

Consider a plant composed by two coupled tanks, where the values of the physical parameters were borrowed from the Coupled Tanks System model 33-041 [49]. The plant diagram is depicted in Figure 3a, where the actuators and sensors are highlighted, and the actual plant from which the physical parameters were obtained is shown in Figure 3b.

Observe that the plant consists in two indistinguishable tanks coupled by an opening valve ($C_{c}$). Variable pace pumps ($Q_{in}^{1}$ and $Q_{in}^{2}$) supply water to the first and second tanks. The hole between the tanks ($C_{c}$) allows the water to stream into the second tank and thus out to a store. The control action aims to adjust the inlet flow rate ($Q_{in}^{1}$ and $Q_{in}^{2}$) in order to maintain the levels $H_{1}$ and $H_{2}$ in the first and second tanks close to some desired set points. The continuous-time dynamic equations that describe the level variation for each tank are given by

(21) $$\begin{array}{c} \frac{\partial H_{1}\left(t\right)}{\partial t}=\frac{KQ_{in}^{1}\left(t\right)}{A_{r}}-\frac{C_{d}a\sqrt{2gH_{1}\left(t\right)}}{A_{r}}-\frac{C_{c}a\sqrt{2g(H_{1}\left(t\right)-H_{2}\left(t\right))}}{A_{r}}+\lambda_{1}\left(t\right),\\ \frac{\partial H_{2}\left(t\right)}{\partial t}=\frac{KQ_{in}^{2}\left(t\right)}{A_{r}}-\frac{C_{d}a\sqrt{2gH_{2}\left(t\right)}}{A_{r}}+\frac{C_{c}a\sqrt{2g(H_{1}\left(t\right)-H_{2}\left(t\right))}}{A_{r}}+\lambda_{2}\left(t\right), \end{array}$$

where $\lambda_{1}$ and $\lambda_{2}$ denote exogenous inputs with null mean and standard deviation given, respectively, by $\rho_{1}$ and $\rho_{2}$. The plant parameters are presented in Table 3.

Adopting the same procedure provided in [50], the state vector is defined as $x\left(t\right)={\left[H_{1}\left(t\right)\;H_{2}\left(t\right)\right]}^{\prime }$, and $\dot{x}\left(t\right)={\left[\Delta H_{1}\left(t\right)\;\Delta H_{2}\left(t\right)\right]}^{\prime }$, where $\Delta H_{1}\left(t\right)$ and $\Delta H_{2}\left(t\right)$ represent the variation of the fluid height around the linearization points of tanks 1 and 2, respectively. Then, by linearizing Equation (21) around $H_{1}=20\;\mathrm{cm}$ and $H_{2}=10\;\mathrm{cm}$, the state-space matrices of a continuous-time system similar to Equation (1) are given by

(22) $$\begin{array}{c} A=\left[\begin{array}{cc} -0.0239 & -0.0127\\ 0.0127 & -0.0285 \end{array}\right],\;E=\left[\begin{array}{cc} 3 & 0\\ 0 & 2.5 \end{array}\right],\;B=0.7198I,\;C_{z}=C_{y}=I,\;E_{y}=D_{z}=E_{z}=0, \end{array}$$

**Figure 4:**
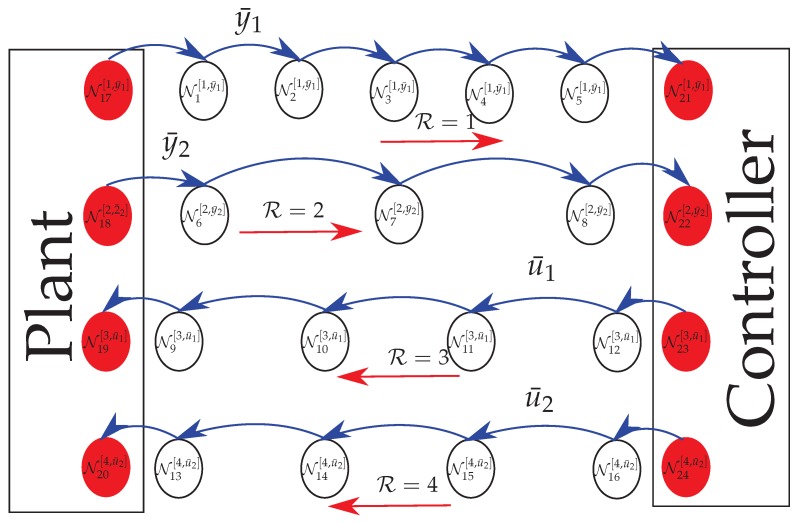
Network topology for the experiment presented in Section 6.

The measurement information required for feedback purposes and the control signal are transmitted, respectively, from the plant to the remote controller and from the controller to the plant in periodic samples (with a sampling interval $T_{S}$) of the output and control signals, through a semi-reliable network with topology shown in Figure 4. To obtain a discrete-model, suitable for digital control, a discretization procedure based on the Zero-Order Holder was implemented. Since the sampling time $T_{S}$ is one of the parameters used to compute the energy consumption cost given by Equation (17), the choice of $T_{S}$ is significant. For instance, choosing an unsuitable value of $T_{S}$ may lead to a discrete-time model that does not accurately represent the plant and, as a consequence, affecting the performance of the controller. An appropriate choice of the sampling interval can be accomplished, for instance, by analyzing the frequency response and computing the rising time [51] Chapter 13, and the bandwidth of the system [52] Chapter 11. Based on the criterion mentioned in [53] Chapters 8 and 9, a recommended sampling time for level tank control is $T_{S}\in \left[5,\;10\right]$ s. Thus, since the value of $T_{S}$ is inversely proportional to the minimization of the energy consumption (see Equation (17)), the sampling time chosen in this experiment was $T_{S}=10$ s.

The measurement vector $y\left(k\right)\in R^{2}$ has two packets associated with independent communication level sensors: ${\bar{y}}_{1}\left(k\right)=H_{1}\left(k\right)$ and ${\bar{y}}_{2}\left(k\right)=H_{2}\left(k\right)$. Regarding the control signal $u\left(k\right)\in R^{2}$, two packets associated with two independent actuators are considered: ${\bar{u}}_{1}\left(k\right)$ and ${\bar{u}}_{2}\left(k\right)$, respectively, representing the control inputs in the first and second tanks. Using the semi-reliable network modeling developed in Section 4.2, one has $n_{\bar{u}}=n_{\bar{y}}=2$ and $\sigma_{u}=\sigma_{y}=2^{2}=4$; two partial transition matrices $P^{u}$ and $P^{y}\in R^{4\times 4}$; and, consequently, the transition matrix that governs the jumps among all modes is given by $P=P^{u}⊗P^{y}\in R^{16\times 16}$. Furthermore, it is assumed that only the modes associated to y(k) are accessible and, as a consequence, the 16 operational modes are organized in four clusters $\sigma_{y}=4$ in function of $P^{y}$, and the partially mode-dependent controller (13) is designed such that $i\in [1,\ldots ,\sigma_{y}]$.

Concerning the communication failure, the Zero-input approach [54,55,56] is adopted in this experiment, that is, the control matrices ($B_{i},D_{z_{i}}$) or the output matrices ($C_{y_{i}}$ and $E_{y_{i}}$) of system (1) are equal to zero in the operation modes i∈K associated to failures. An example of application of this approach in dynamic output-feedback control problem can be seen in [57,58]. Besides solving the tank level control problem by using Lemmas 2 and 3, the proposed Energy-Efficiency Protocol optimizes the energy consumption of the network by limiting the maximum number of allowed transmissions of the network nodes and, at the same time, assuring the closed-loop stability and providing an acceptable $H_{\infty }$ closed-loop performance.

### 6.2. Network Parameters

In this experiment, it is assumed that the measurement information gathered by each sensor and the control signal associated with each actuator are transported separately, as illustrated in Figure 4.

The first route R=1, which has seven nodes (including the source and sink nodes), transports the information referent to ${\bar{y}}_{1}\left(k\right)=H_{1}\left(k\right)$. This route has N=6 jumps, and each one has success probability equal to $p({\bar{y}}_{1})=0.5$. The second route R=2 transports the fragment $y_{2}=H_{2}\left(k\right)$ and has N=4 jumps and five nodes, considering the source and sink nodes, such that each node has the success probability equal to $p({\bar{y}}_{2})=0.5$. On the other hand, the transmission of the control signal packets ${\bar{u}}_{1}\left(k\right)$ and ${\bar{u}}_{2}\left(k\right)$, is made, respectively, by routes R=3 and R=4, both having six nodes, considering source and sink nodes, implying N=5 jumps. The success probability for each node of route R=3 is $p({\bar{u}}_{1})=0.45$, while for route R=4 is $p({\bar{u}}_{2})=0.4$. All the network parameters are summarized in Table 4.

As proposed in Section 5.5, the following criteria are used to evaluate the Protocol of Energy-Efficiency of the communication network:

- The global energy consumption (quantified in J/hour) computed by
  (23) $$E_{GE}=\sum_{R\in T_{R}}E\left(E_{T}\left(p,L,N\right)\right)\left(R\right)$$
  with $E(E_{T}\left(p,L,N\right))$ given in Equation (17);
- The energy consumed per unit of time $E|M^{\left(i\right)}|$ by the node *U* with the highest data traffic (number of transmissions) in the network. The value of $E|M^{\left(i\right)}|$ can be computed using the methodology proposed in [6] for i=1,…,N, where *N* is the number of hops of the route, or using the R Package ‘Hopbyhop’ [43], as shown in Appendix A.

It is important to emphasize that the source and sink nodes are neglected in the determination of the critical node (the one with the highest consumption), since it is considered that they are associated with sensors and actuators with their own batteries. Thus, their energy consumption for communication purposes is not relevant. Besides the sampling period $T_{S}=10$ s, the parameters required for the computation of Equation (17) are information available in actual communication networks which, in this particular example, were obtained from (The actual energy consumption parameters are based on XBee Pro devices [59]) [9] and [13] Section 7.3.1, corresponding to: $E_{TX}=228$ μJ, $E_{RX}=208$ μJ, $E_{SW}=0$, Ξ=3600.

### 6.3. Results

For comparison purposes, it is first designed a dynamic output-feedback controller using the classic control approach (no packet loss, L=∞, that is, $P_{S}=1$ in Equation (15) for all routes). Applying this assumption in Lemmas 2 and 3 for the design of the controller, an $H_{\infty }$ guaranteed cost of 63.2076 (computed through Lemma 1 is obtained for the closed-loop system (13). Additionally, for a full-reliable communication, the mathematical expectation of the global number of transmission E(M) for each route $R_{1}$, $R_{2}$, $R_{3}$ and $R_{4}$ is, respectively, 36, 24, 35.8025 and 43.75. The value of E(M) is computed for each route with L=∞, *p* and *N* given in Table 4. Consequently, the energy consumed by route (according to Equation (17)) is, respectively, 4.3027 J/h, 2.8685 J/h, 4.1451 J/h and 4.9014 J/h, and the global energy consumption is 16.2177 J/h.

#### 6.3.1. Energy Saving Protocol—Trade-Off Approach—*L* Unique

**Figure 5:**
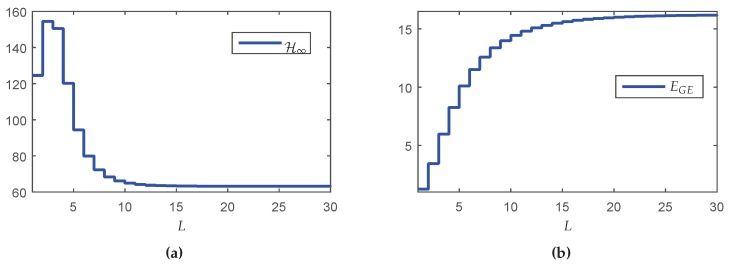
(**a**) $H_{\infty }$ guaranteed costs. (**b**) Global energy consumption versus *L* for the Trade-off approach.

In this section, a dynamic output-feedback controller is designed considering the case where *L* is fixed ∈[1,…,30]. The maximum value of *L* was chosen equal to 30 because the probability of successful transmission between the source and sink ($P_{S}$) for each fragment of y(k) and u(k) tends to 1 for L>30, which provides the same performance of the classic control design. The minimum value of *L* can not be zero, since in this case the system would operate in open-loop (null control signal). Thus, the minimum value of *L* was set to L=1, ensuring a closed-loop operation. Using these assumptions in Lemmas 2 and 3 for the design of the Markovian controller, one has the results depicted in Figure 5a, which shows the $H_{\infty }$ guaranteed costs of the closed-loop system in terms of the maximum number of transmissions *L*.

Although counterintuitive, note that the performance in terms of the $H_{\infty }$ norm for L=1 is better than the result for L=3. A possible reason is that the synthesis of the controller was made in two steps (observer design + state-feedback control design). Although each step of design provides the optimal result, even for the partially mode-dependent and mode-independent cases, the joint behavior is not necessarily optimal (conservative). On the other hand, the global energy consumption increases monotonically as *L* grows (see Figure 5b), meaning that any reduction of *L* implies in resource saving. To evidence how this saving occurs, Table 5 shows some performance measurements variables (defined in Section 5.1). Besides the performance indexes $\Omega_{H}$, $\Omega_{M}$, $Y_{H}$, $Y_{M}$ and $H_{\infty }$ norm, it is also presented $E_{GE}\left(N_{max}\right)$ and $\Omega_{E}\left(N_{max}\right)$, representing the criteria computed in the node with highest consumption ($N_{max}$). In this example, the first node (without considering the Source and sink) was the worst one.

Observe that when L=8, the increment in the $H_{\infty }$ norm in comparison with the classical case is 8.07%, however, the increment in the energy saving is about twice bigger: 17.37%. On the other hand, for L=5 it is possible to obtain a more expressive energy saving: 37.58%, nevertheless the $H_{\infty }$ norm is 49.25% greater than the classical case. Although L=5 provides greater energy savings, the trade-off relationship between the norm and energy savings is negative. Even so, this choice can be attractive depending on the criterion adopted by the designer.

Knowing that the node with highest energy consumption is the node closest to the source belonging to the route with the lowest probability per hop (p=0.4), one has that the critical node is $N_{16}^{\left[4,{\bar{u}}_{2}\right]}$. Another information obtained from Table 5 is that the variation in the global energy consumption ($E_{GE}$) in function of *L* does not present a linear relationship with the variation in the energy consumption of the critical node ($E_{GE}\left(N_{16}^{\left[4,{\bar{u}}_{2}\right]}\right)$).

#### 6.3.2. Energy Saving Protoco—Second Method: *L* Variable

In the first method, the value of *L* is fixed and the resulting performance, for instance the robustness against exogenous inputs, is always worse than the one provided by the classic case. In the second method, the value (per node) of $L\in \{L_{1},\;L_{2}\}$ depends exclusively on the battery energy levels, which are time-varying. In the network presented in Figure 4, the nodes that change their maximum number of transmissions *L* over time are U∈{1,2,…,16}, while nodes U∈{17,18,…,24} always operate in $L_{1}=\infty$ because they (nodes connected to the sensors and actuators) are assumed to have unlimited energy. The proposed method starts with a full-reliable communication (L=∞), providing the best performance in terms of $H_{\infty }$ norm. The vector $L=\{L_{1}=\infty ,L_{2}=5\}$ starts with a norm value of $H_{\infty }=63.2076$ and an global energy consumption for the entire network equal to 16.2177 J and gradually declines to the value of L=5 for all nodes with a performance of $H_{\infty }=88.2573$ and a energy consumption 11.5137 J/h.

Table 6 exhibits the probabilities of successful transmission between the source and sink (Pr$\left({\bar{y}}_{1}\right)$, Pr$\left({\bar{y}}_{2}\right)$, Pr$\left({\bar{u}}_{1}\right)$, and Pr$\left({\bar{u}}_{2}\right)$) associated with each measurement or control input signal ($\left({\bar{y}}_{1}\right)$, $\left({\bar{y}}_{2}\right)$, $\left({\bar{u}}_{1}\right)$, and $\left({\bar{u}}_{2}\right)$) for each new setting of the network. More precisely, Table 6 shows the sequence where the nodes change from $L_{1}=\infty$ to $L_{2}=5$, such that column *U* indicates the node that is changing in the current time interval and column $I_{L_{m}}$ indicates the order of each setting in terms of P(ξ).

By imposing a one-way exchange sequence (from $L_{1}=\infty$ to $L_{2}=5$ and never the opposite), the network under investigation can operate only in ξ=17 distinct settings, such that the change in the value of *L* is made via an alarm signal sent to the controller. Observe that the exchange sequence by node is supposedly done according to the battery consumption of each node (The selection of the switching sequence is merely illustrative. To determine an optimized switching sequence for a real network is outside the scope of investigation of the present work).

**Figure 6:**
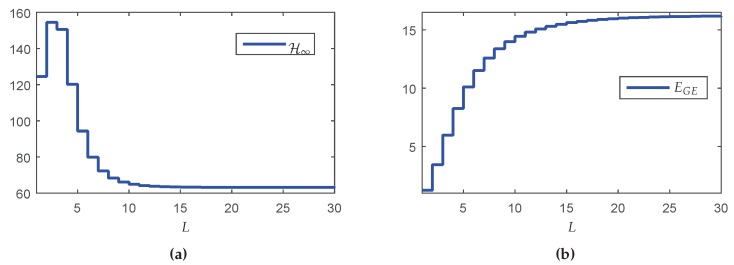
(**a**) $H_{\infty }$ norm of the closed-loop system (**b**) Expected number of global transmissions by route (E(M)) (**c**) Global energy consumption of the network ($E_{GE}$) computed for each setting ξ of the network associated with the exchange sequence $I_{Lm}$.

Figure 6a shows the $H_{\infty }$ guaranteed costs of the closed-loop system computed for each transition probability matrix P(ξ) associated to a new network setting ξ=[1,17] respecting the exchange order indicated by $I_{L_{m}}$ in Table 6. Note that the guaranteed costs do not increase monotonically. As explained before, the reason for this fact is that the design conditions are not optimal, that is, the performance index is not directly associated with the increase of the probabilities. Figure 6b shows the values of the mathematical expectation of the global number of transmissions E(M) by route computed according with Lemma 4. Since the greater the number of transmissions, the greater the energy consumption, observe that the route associated with the highest resource consumption is $R_{4}$, that also contains the critical network node. Finally, Figure 6c exhibits the energy consumption $E_{GE}$ for each exchange interval $I_{L_{m}}$, that is monotonic decreasing, meaning that the greater the number of devices operating in the resource savings working mode (finite *L*), the greater are the savings in the global energy consumption of the network.

As conclusion, note that the main advantage of this method is that, at first, the performance is optimized (minimum in terms of $H_{\infty }$ norm, that is equivalent to the classical case since L=∞) and over time, although the $H_{\infty }$ performance is deteriorated, the energy saving is gradually increased, augmenting the equipment life span.

#### 6.3.3. Energy Saving Protoco—Third Method: Mixed Problem

**Figure 7:**
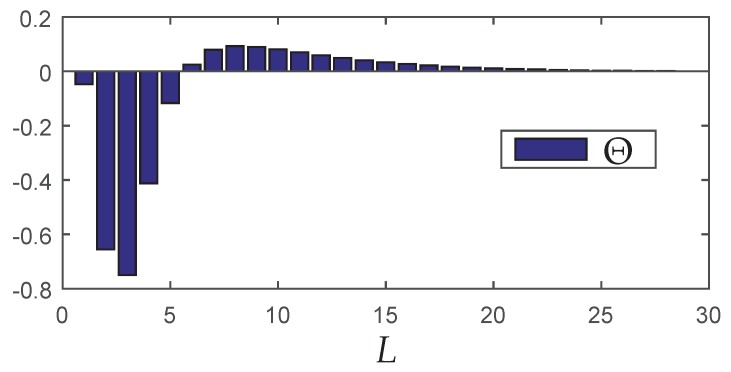
Performance measurement index Θ in terms of a fixed *L* for the network under investigation.

Although associated with the optimal performance in terms of the $H_{\infty }$ norm, the implementation of a full-reliable communication is unfavorable with respect the energy consumption. Depending on the network and the dynamic system to be controlled, to allow a small packet loss percentage may provide a significant decrease in the use of network resources and a proportionally smaller decrease in the performance. A similar problem was discussed in [6,7,8,13], concerning filtering design through semi-reliable networks. The main goal in those works is to keep a positive trade-off between the norm degradation and the energy savings, denoted by Θ and defined in Section 5.1 of this paper. To employ the mixed energy saving protocol approach, first it is necessary to determine the initial finite value of *L*. By setting the maximum number of transmissions of all the network nodes to the same fixed and finite value of *L*, it is possible to compute the resulting value of Θ (based on the results of Section 6.3.1, see Figure 7). Since the mixed approach does not include the case $L_{1}=\infty$ and aiming to use values associated with positive trade-off, two distinct finite values of *L* were chosen: $L_{2}=8$ and $L_{3}=5$, that is $L\in \left\{L_{2},L_{3}\right\}=\left\{8,5\right\}$. The initial value was chosen L=8 since it results in the maximum trade-off Θ, and the energy savings work mode was fixed in L=5 to allow a better comparison with the second approach, where the finite maximum number of transmissions was also 5. To compute the number of distinct network settings (in this case, the same as the previous method: ξ=17, as shown in Table 7), first it is necessary to identify which nodes change the maximum number of transmissions *L* over time (U∈{1,2,…,16}), and which nodes always operate in fixed L=8 (U∈{17,18,…,24}) because they are connected to the sensors and actuators with unlimited energy.

**Figure 8:**
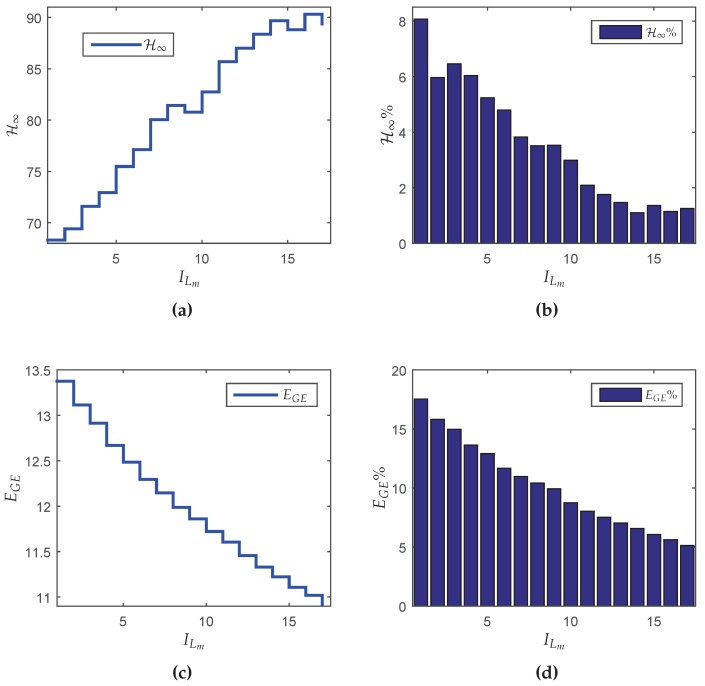
(**a**) $H_{\infty }$ norm of the closed-loop system using the mixed approach; (**b**) Percentage difference between the norm obtained by the second and third energy saving protocols; (**c**) The global energy consumption ($E_{GE}$) of the network using the mixed approach; (**d**) Percentage difference between the global energy consumptions obtained by the second and third energy saving protocols. All the parameters are evaluated in terms of the distinct network settings organized according to the sequence $I_{Lm}$.

Figure 8 shows the results of the third method in terms of the $H_{\infty }$ performance and the global energy consumption $E_{GE}$, and also a comparison with the results obtained by the second approach. Observe, for instance, that for $I_{L_{m}}=1$, all the nodes of the network are using L=8, corresponding to the performance presented in Table 5 for column L=8. Similarly to the second method, Figure 8a shows that the $H_{\infty }$ norm does not increase monotonically. Figure 8b illustrates the percentage augmentation of the $H_{\infty }$ norm for each network setting when compared with the value obtained by the second method:

$$H_{\infty }\%=\frac{H_{\infty }^{A3}\left(I_{L_{m}}\right)-H_{\infty }^{A2}\left(I_{L_{m}}\right)}{H_{\infty }^{A2}\left(I_{L_{m}}\right)}\times 100,$$

where $H_{\infty }^{A2}\left(I_{L_{m}}\right)$ and $H_{\infty }^{A3}\left(I_{L_{m}}\right)$ respectively represent the $H_{\infty }$ norm of the closed-loop system computed by the second approach (energy by node approach) and the third approach (mixed approach) for each network setting indexed by $I_{L_{m}}$.

On the other hand, Figure 8c shows that the global energy consumption $E_{GE}$ decreases monotonically with the augmentation of the number of devices operating in the energy saving work mode. Analogously to what was done for the $H_{\infty }$ norm, Figure 8b illustrates the percentage augmentation of $E_{GE}$ for each network setting when compared with the value obtained by the second method:

$$E_{GE}\%=\frac{E_{GE}^{A3}\left(I_{L_{m}}\right)-E_{GE}^{A2}\left(I_{L_{m}}\right)}{E_{GE}^{A2}\left(I_{L_{m}}\right)}\times 100,$$

where $E_{GE}^{A2}\left(I_{L_{m}}\right)$ and $E_{GE}^{A3}\left(I_{L_{m}}\right)$ respectively represent the global energy consumption associated with the second approach (energy by node approach) and with the third approach (mixed approach) for each network setting indexed by $I_{L_{m}}$.

#### 6.3.4. Energy Saving Protocol: Application Guidelines and the Comparison of the Three Approaches

Before comparing the performance and energy consumption provided by the three proposed methods, a summarized sequence of operations that must be performed to apply the proposed energy saving protocol is described, step-by-step, in what follows.

1. The output-feedback controller is designed considering the classical control theory (full-reliable network, L=∞, $P_{S}=1$) and the optimal value for the $H_{\infty }$ norm of the closed-loop system is calculated;
2. The designer chooses a maximum value for the $H_{\infty }$ norm degradation (when compared with the optimum $H_{\infty }$ norm computed in step 1: $Y_{H}$), which may be considered acceptable within the project specifications;
3. Consider the implementation of the first and most simple heuristic procedure of energy saving protocol: Trade-off approach (*L* fixed and unique), named A1 in this section. Choose a maximum number of allowed transmissions (*L*) and determine the associated transition probability matrix to finally compute the controllers and the $H_{\infty }$ closed-loop norm (using Lemmas 2 and 3) and the network global energy consumption ($E_{GE}$ from Equation (23));
4. If the $H_{\infty }$ norm degradation (rate between the norm obtained with packet loss and the one using the classical approach, without loss, $Y_{H}$) satisfies the performance required by the designer, it is possible to reduce the value of *L*, implying a more accentuated reduction in the energy consumption. Otherwise, a value of *L* greater than the previous one must be chosen (increasing the network reliability and consequently the probability of successful transmissions) to satisfy the maximum norm degradation determined by the designer in step 2;
5. Assuming the new value of *L* set in step 4, the third and fourth steps are repeated such that the maximum number of allowed transmissions converge to a minimum value ($L_{min}$) that simultaneously respects the norm degradation criterion and maximizes the energy savings (best trade-off Θ achieved).

Considering the implementation of the second heuristic method of energy saving protocol (*L* variable from *∞* to a fixed and finite value), named A2 in this section, it is known, from the results obtained for A1 (steps 1 to 5), the minimum value of *L* common to all network nodes which does not violate the criterion of norm degradation imposed by the designer. Also knowing that in the second heuristic (A2), each network node assumes *a priori* the value L=∞ and then the maximum number of allowed transmissions converges to $L=L_{min}$ (except the nodes connected to the sensors or actuators, red circles in Figure 4), it is enough to compute all the different combinations of network settings (Table 6), and the corresponding transition probability matrices, stabilizing controllers, $H_{\infty }$ closed-loop norm (Figure 6a), norm degradation $Y_{H}$, and network global energy consumption (Figure 6c). Observe that A2 can be less efficient in terms of energy consumption than A1 because the network nodes operate, during a certain time-window, in full-reliable working mode. However, it is more efficient in terms of $H_{\infty }$ performance because the network nodes operate, during a certain time-window, close to the optimal solution.

Finally, for the implementation of the third heuristic method of energy saving protocol (*L* variable inside a set of values fixed and finite), named A3 in this section, besides determining a maximum value for the $H_{\infty }$ norm degradation (as done for A1 and A2), the designer must also set the maximum global energy consumption by choosing an upper bound for the maximum number of allowed transmissions for each node ($L_{max}$). This value of *L* can be chosen based on the results of energy consumption obtained for A1, since the network starts its operation in a certain configuration with a finite and fixed value of *L* common to all nodes, such that it does not violate the norm degradation criterion imposed by the designer, and also allows an acceptable $H_{\infty }$ performance. Similarly to A2, knowing that each node assumes *a priori* the value $L=L_{max}$ and then converges to $L=L_{min}$ (except the nodes connected to the sensors or actuators, red circles in Figure 4, that remain fixed in $L_{max}$), it is sufficient to compute all the different combinations of network settings (Table 7), and the corresponding transition probability matrices, stabilizing controllers, $H_{\infty }$ closed-loop norm (Figure 8a), norm degradation $Y_{H}$, and network global energy consumption (Figure 8c).

**Figure 9:**
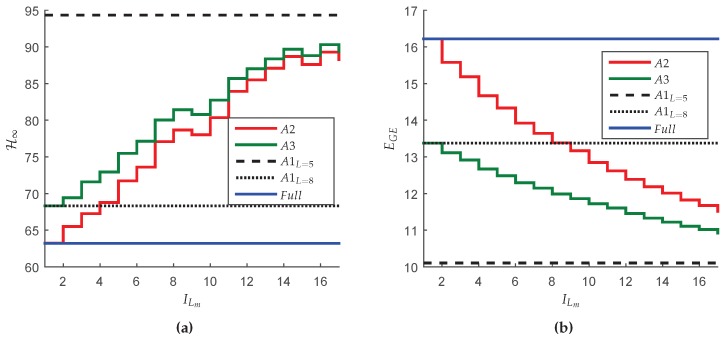
(**a**) $H_{\infty }$ norm of the closed-loop system and (**b**) The global energy consumption ($E_{GE}$) of the network using: the classical approach assuming a full-reliable (L=∞) network (in blue); A1 with L=5 ($A1_{L=5}$ in dashed black); A1 with L=8 ($A1_{L=8}$ in dotted black); A2 with L∈{∞,5} (A2 in red); A3 with L∈{8,5} (A3 in green).

Aiming to compare both performance and energy consumption of all three approaches and their advantages with respect to the classical approach (that assumes the unrealistic hypothesis of a full-reliable network), the $H_{\infty }$ closed-loop norm and the value of $E_{GE}$ obtained with each one of the methods are presented in Figure 9. Observe in Figure 9a that, as expected, the $H_{\infty }$ norm associated with the classical approach is the optimal one and, therefore, an lower bound for the second approach ($A_{2}$ with L∈{∞,5}), since the starting setting of the network imposes L=∞ for all nodes. Also note that, the $H_{\infty }$ performance obtained for the first method (A1) with L=8 is a lower bound for the third approach ($A_{3}$ with L∈{8,5}), since, in this case, all the network nodes are set in L=8 in the starting setting. Furthermore, the $H_{\infty }$ results for A1 with L=5 are always superior than those obtained with any other method, because, even for $A_{2}$ with L∈{∞,5} and $A_{3}$ with L∈{8,5}, the nodes connected to sensors and actuators are assumed operating with unlimited energy (L=∞ for A2 and L=8 for A3). The same explanation can be used to justify why the $H_{\infty }$ closed-loop norm provided by A2 is always better than the values provided by A3 even if all the intermediate nodes converge to L=5. On the other hand, it is noteworthy to mention that the features that can classify a given method as unfavorable in terms of $H_{\infty }$ performance are the same that allow greater energy savings (see Figure 9b). Note, for instance, that A3 tends to save less energy than A1 with L=5 (because the network nodes operate, for a certain time-window, in a more reliable working mode: L=8) but it consumes less energy than A2 (because no network node operates in full-reliable working mode when using A3).

**Figure 10:**
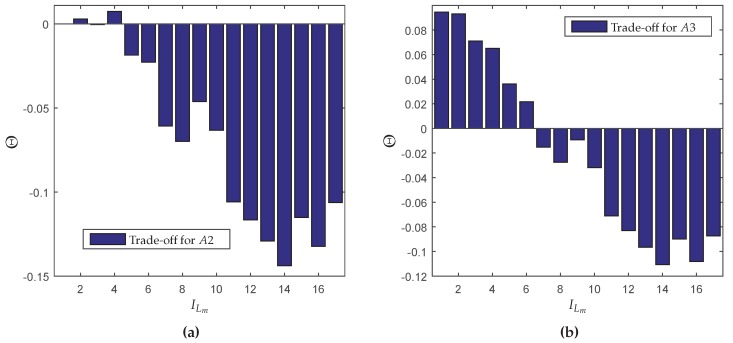
Trade-off (Θ) of the system evaluated in terms of the distinct network settings ξ according with $I_{Lm}$ for: (**a**) the second approach (A2 with L∈{∞,5}) and (**b**) the third approach (A3 with L∈{8,5}).

To evaluate how much better is A3 with L∈{8,5} than A2 with L∈{∞,5} or A1 with L=5, the trade-off index (Θ) is presented in Figure 10 for each distinct network setting indexed by $I_{L_{m}}$. First, note that by employing A1 with L=5, the trade-off is negative (see Figure 7). By employing A2, there are only two network settings that present a positive trade-off, since, in a large time-window, several nodes are maintained in full-reliable working mode. On the other hand, when using A3, there are six network settings that present a positive trade-off and even when negative, the trade-off associated to A3 is better than the other methods.

In short, note that the third approach always produces better results in terms of energy savings than the second method, which starts from the setting similar to the classical control case. Furthermore, when compared with the first method, the third approach seems to be more flexible, allowing to operate on distinct energy saving working modes, among which there are some with better $H_{\infty }$ performance indexes.

## 7. Conclusions

This paper handled the problem of energy efficiency in NCS scenario. To perform the design of a stabilizing dynamic output-feedback controller connected through a semi-reliable communication network to the plant, a discrete-time representation based on MJLS is proposed. The synthesis of the controller is made in two steps: (i) the design of a state-observer and (ii) the design of a state-feedback controller. Although both steps provide optimal solutions individually, the joint procedure provides a sub-optimal solution. The first contribution of the paper is the modeling of the Makov chain in terms of the packets that group the information about the control or measurement output signals, such that the packet loss or the successful transmission of those packets affect the accessibility of the chain modes and the computation of the global transition probability matrix of the MJLS. The second contribution is to evaluate the behavior of the network global energy consumption levels associated to three distinct energy saving protocols, which are defined in terms of the maximum number of transmissions allowed by the nodes (*L*). The first method uses a single, fixed and finite value of *L*, implying a closed-loop system with degraded performance but energy consumption levels lower than the classical case. The second approach employs a finite set of values of *L*, starting from the case without packet loss (L=∞), such that the initial $H_{\infty }$ performance is similar to the one obtained by the classical control case, and then it switches to a set of finite values of *L*, generating a group of energy saving working modes. The third technique join the advantages of the two previous approaches: as the first method, it always operates in a energy saving working mode (when compared with the classical control case), however, as done in the second approach, it generates a set of distinct working modes where the resource saving can compensate the performance degradation. Summarizing, as shown in the simulation results obtained for the control of a linearized model of a coupled multi-level tank through a semi-reliable communication network with a particular topology, all three approaches are suitable solutions to achieve some energy efficiency in the NCS problem. Each one of them may be more suitable than the others depending on the controlled system, on the network topology or the designer priorities.

## Figures and Tables

**Table 1:** Probability of each mode in $\ell_{y}$.

$\ell_{y}$	${\bar{y}}_{1}$	${\bar{y}}_{2}$	$\mathrm{Pr}(\ell_{y})$
1	ok	ok	$P_{{\bar{y}}_{1}}P_{{\bar{y}}_{2}}$
2	ok	error	$P_{{\bar{y}}_{1}}\left(1-P_{{\bar{y}}_{2}}\right)$
3	error	ok	$\left(1-P_{{\bar{y}}_{1}}\right)P_{{\bar{y}}_{2}}$
4	error	error	$\left(1-P_{{\bar{y}}_{1}}\right)\left(1-P_{{\bar{y}}_{2}}\right)$

**Table 2:** Probability of each mode in $\ell_{u}$.

$\ell_{u}$	${\bar{u}}_{1}$	${\bar{u}}_{2}$	$\mathrm{Pr}(\ell_{u})$
1	ok	ok	$P_{{\bar{u}}_{1}}P_{{\bar{u}}_{2}}$
2	ok	error	$P_{{\bar{u}}_{1}}\left(1-P_{{\bar{u}}_{2}}\right)$
3	error	ok	$\left(1-P_{{\bar{u}}_{1}}\right)P_{{\bar{u}}_{2}}$
4	error	error	$\left(1-P_{{\bar{u}}_{1}}\right)\left(1-P_{{\bar{u}}_{2}}\right)$

**Table 3:** Plant parameters used in the example.

*g*	9.8 $m/s^{2}$	gravitational acceleration
$A_{r}$	138.92 ${\mathrm{cm}}^{2}$	the cross-section area of the tanks
*a*	3.14 ${\mathrm{cm}}^{2}$	the cross-section area of the pipe connecting the two tanks
$C_{d}$	1	the valve discharge coefficient
$C_{c}$	0.8	the valve discharge coefficient of the coupled tanks
$\lambda_{1}$	3 cm	the disturbance at the tank level 1
$\lambda_{2}$	2.5 cm	the disturbance at the tank level 2
$Q_{in}^{1}$	$u_{1}\left(t\right)$	the input flow of the liquid in tank 1
$Q_{in}^{2}$	$u_{2}\left(t\right)$	the input flow of the liquid in tank 2
K	100	gain associated to a change of scale

**Table 4:** Network parameters used in the experiment.

R	*p*	*N*	S
1	0.5	6	${\bar{y}}_{1}$
2	0.5	4	${\bar{y}}_{2}$
3	0.45	5	${\bar{u}}_{1}$
4	0.4	5	${\bar{u}}_{2}$

**Table 5:** Performance measurement indexes of the network for a single *L*.

***L***	**1**	**2**	**3**	**4**	**5**	**6**	**7**	**8**	**9**	**10**
$H_{\infty }$	124.5475	154.4460	150.5228	120.2178	94.3426	79.8638	72.2903	68.3102	66.1161	64.8694
E(M)	10.6628	29.3899	51.0620	70.7259	86.5859	98.7444	107.9099	114.8178	120.0624	124.0839
$E_{GE}$	1.2474	3.4379	5.9685	8.2596	10.1035	11.5141	12.5757	13.3745	13.9802	14.4440
$\Omega_{H}$	0.9705	1.4435	1.3814	0.9020	0.4926	0.2635	0.1437	0.0807	0.0460	0.0263
$\Omega_{E}$	0.9229	0.7876	0.6312	0.4897	0.3758	0.2886	0.2230	0.1737	0.1363	0.1076
$E_{GE}\left(N_{max}\right)$	0.1076	0.2475	0.3834	0.5006	0.6039	0.6876	0.7563	0.8128	0.8595	0.8985
$\Omega_{E}\left(N_{max}\right)$	0.9011	0.7730	0.6487	0.5415	0.4471	0.3705	0.3077	0.2560	0.2133	0.1776
***L***	**11**	**12**	**13**	**14**	**15**	**16**	**17**	**18**	**19**	**20**
$H_{\infty }$	64.1627	63.7602	63.5295	63.3959	63.3179	63.2721	63.2452	63.2294	63.2200	63.2145
E(M)	127.1993	129.6355	131.5560	133.0806	134.2979	135.2745	136.0613	136.6974	137.2131	137.6325
$E_{GE}$	14.8028	15.0831	15.3038	15.4788	15.6184	15.7303	15.8203	15.8930	15.9519	15.9997
$\Omega_{H}$	0.0151	0.0088	0.0051	0.0030	0.0018	0.0010	0.0006	0.0004	0.0002	0.0001
$\Omega_{E}$	0.0854	0.0681	0.0545	0.0437	0.0350	0.0281	0.0226	0.0181	0.0144	0.0115
$E_{GE}\left(N_{max}\right)$	0.9299	0.9564	0.9786	0.9968	1.0123	1.0252	1.0361	1.0452	1.0528	1.0592
$\Omega_{E}\left(N_{max}\right)$	0.1489	0.1247	0.1044	0.0876	0.0735	0.0617	0.0517	0.0434	0.0364	0.0305
***L***	**21**	**22**	**23**	**24**	**25**	**26**	**27**	**28**	**29**	**30**
$H_{\infty }\times 10^{3}$	63.2112	63.2092	63.2081	63.2074	63.2070	63.2067	63.2066	63.2065	63.2064	63.2064
E(M)	137.9742	138.2532	138.4815	138.6687	138.8223	138.9485	139.0525	139.1382	139.2089	139.2673
$E_{GE}$	16.0386	16.0704	16.0963	16.1176	16.1351	16.1494	16.1612	16.1709	16.1789	16.1855
$\Omega_{H}$	0.0763	0.0455	0.0271	0.0161	0.0094	0.0054	0.0030	0.0015	0.0006	0.00
$\Omega_{E}$	0.0091	0.0071	0.0055	0.0042	0.0031	0.0022	0.0015	0.0009	0.0004	0.00
$E_{GE}\left(N_{max}\right)$	1.0645	1.0697	1.0734	1.0765	1.0786	1.0808	1.0826	1.0843	1.0856	1.0868
$\Omega_{E}\left(N_{max}\right)$	0.0257	0.0209	0.0176	0.0148	0.0128	0.0108	0.0092	0.0076	0.0064	0.0053

**Table 6:** Probabilities of successful transmission for each new setting of the network such that the nodes *U* exchange L∈{∞,5} following the sequence $I_{Lm}$ (Energy for node approach).

$I_{L_{m}}$	*U*	Pr$\left({\bar{y}}_{1}\right)$	Pr$\left({\bar{y}}_{2}\right)$	Pr$\left({\bar{u}}_{1}\right)$	Pr$\left({\bar{u}}_{2}\right)$
1	−	1	1	1	1
2	13	1.0000	1.0000	1.0000	0.9222
3	9	1.0000	1.0000	0.9497	0.9222
4	14	1.0000	1.0000	0.9497	0.8505
5	10	1.0000	1.0000	0.9019	0.8505
6	15	1.0000	1.0000	0.9019	0.7844
7	11	1.0000	1.0000	0.8565	0.7844
8	1	0.9688	1.0000	0.8565	0.7844
9	6	0.9688	0.9688	0.8565	0.7844
10	16	0.9688	0.9688	0.8565	0.7234
11	12	0.9688	0.9688	0.8134	0.7234
12	2	0.9385	0.9688	0.8134	0.7234
13	3	0.9091	0.9688	0.8134	0.7234
14	4	0.8807	0.9688	0.8134	0.7234
15	7	0.8807	0.9385	0.8134	0.7234
16	5	0.8532	0.9385	0.8134	0.7234
17	8	0.8532	0.9091	0.8134	0.7234

**Table 7:** Probabilities of successful transmission for each new setting of the network such that the nodes *U* exchange L∈{8,5} following the sequence $I_{Lm}$ (Mixed approach).

$I_{L_{m}}$	*U*	Pr$\left({\bar{y}}_{1}\right)$	Pr$\left({\bar{y}}_{2}\right)$	Pr$\left({\bar{u}}_{1}\right)$	Pr$\left({\bar{u}}_{2}\right)$
1	−	0.9768	0.9845	0.9588	0.9188
2	13	0.9768	0.9845	0.9588	0.8618
3	9	0.9768	0.9845	0.9183	0.8618
4	14	0.9768	0.9845	0.9183	0.8084
5	10	0.9768	0.9845	0.8794	0.8084
6	15	0.9768	0.9845	0.8794	0.7583
7	11	0.9768	0.9845	0.8422	0.7583
8	1	0.9500	0.9845	0.8422	0.7583
9	6	0.9500	0.9574	0.8422	0.7583
10	16	0.9500	0.9574	0.8422	0.7112
11	12	0.9500	0.9574	0.8066	0.7112
12	2	0.9239	0.9574	0.8066	0.7112
13	3	0.8985	0.9574	0.8066	0.7112
14	4	0.8739	0.9574	0.8066	0.7112
15	7	0.8739	0.9312	0.8066	0.7112
16	5	0.8499	0.9312	0.8066	0.7112
17	8	0.8499	0.9056	0.8066	0.7112

**Figure A1:**
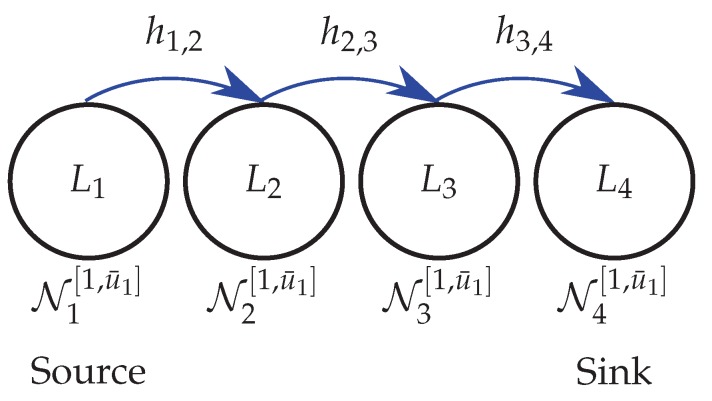
Network for the appendix example.

## Abbreviations

WSN	Wireless Sensor Network
QoS	Quality of Service
LR-WPANs	Low-Rate Wireless Personal Area Networks
NCS	Networked Control Systems
MJLS	Markov Jump Linear System
WLAN	Wireless Local Area Networks
MIMO	Multiple-Input Multiple-Output
EKF	Extended Kalman Filter
ARQ	Automatic Repeat reQuest
ACK	Acknowledgment
TCP	Transmission Control Protocol
CSMA/CA	Carrier Sense Multiple Access with Collision Avoidance
MAC	Media Access Control
FEC	Forward Error Correction
2D-DWT	2-Dimensional Discretized Wavelet Transform
MSS	Mean Square Stability
LMIs	Linear Matrix Inequalities
TX	Transmission Mode
RX	Reception Mode

## Appendix A. R-Package ‘Hopbyhop’ Tutorial

An R-Package named ‘Hopbyhop’ was proposed in order to facilitate the computation of the transmission success probability between source and sink ($P_{\bar{y_{i}}}$ and $P_{\bar{u_{i}}}$ presented in Equations (19) and (20) in Section 4.2.1), and the corresponding values of the expected number of global transmissions in the network by route: E(M). Besides providing all the parameters resulting from Lemma 4, this computational toolbox can calculate individually the expected number of data transmissions and acknowledgment signal transmissions (discussed in [6]). Additionally, with a small change in the function that calculates the parameters of Lemma 4, the package provides numerical results about the mean of the number of transmissions/receptions for Hop-by-Hop model with L-limited retransmissions per packet obtained via Monte Carlo simulations. The manual can be found in [43] and at https://cran.r-project.org/web/packages/hopbyhop/hopbyhop.pdf. Finally, by using the following code in the R console:

> install.packages(“Hopbyhop”)

> library(Hopbyhop)

the download and the installation of the R-package ‘Hopbyhop’ are automatically done.

### Appendix A.1. Full-Reliable and Semi-Reliable Communication

Considering the chain topology presented in Figure A1 with N=3 hops, and supposing that the probability for each hop is p=0.5, the expected global number of transmissions and the probability values (between source and sink) in a full-reliable (unlimited *L*) multi-hop network can be calculated by the R-package ‘Hopbyhop’ using the function.

>  HBH(p1, p2, Inf, N)

where p1=p2=p=0.5, N=3, L=∞. This command provides the following results in the R-console.

###########################################

HOP BY HOP - THEORETICAL RESULTS

###########################################

Data success probability         p1 =  0.5

ACK success probability          p2 =  0.5

Maximum number of transmissions  L  =  Inf

Number of Hops                   N  =  3

Hop 1/3 Hop 2/3 Hop 3/3 Total

Success Probability                1       1       1     1

Expected Data Transmissions        4       4       4    12

Expected ACK Transmissions         2       2       2     6

Expected Total Transmissions       6       6       6    18

Expected Data Receptions           2       2       2     6

Expected ACK Receptions            1       1       1     3

Expected Total Receptions          3       3       3     9

In this paper, the relevant output parameters exhibited in the R-console are: the value of $P_{S}$ from Equation (15), denoted by Success Probability Total, and the value of E(M), represented in the R-console by Expected Total Transmissions.

For the case where the value of *L* is limited and unique, the only change in the command line corresponds to replace Inf by the desired value the *L*. For instance, supposing L=4, one has

> HBH(0.5, 0.5, 4 , 3)

resulting in the following printing in the R console.

###########################################

HOP BY HOP - THEORETICAL RESULTS

###########################################

Data success probability         p1 =  0.5

ACK success probability          p2 =  0.5

Maximum number of transmissions  L  =  4

Number of Hops                   N  =  3

Hop 1/3 Hop 2/3 Hop 3/3  Total

Success Probability            0.938   0.879   0.824  0.824

Expected Data Transmissions    2.734   2.563   2.403  7.701

Expected ACK Transmissions     1.367   1.282   1.202  3.851

Expected Total Transmissions   4.102   3.845   3.605 11.552

Expected Data Receptions       1.367   1.282   1.202  3.851

Expected ACK Receptions        0.684   0.641   0.601  1.925

Expected Total Receptions      2.051   1.923   1.802  5.776

### Appendix A.2. Computation of the Probability of Successful Transmission in A Network with Different Values of L

In the second and third energy saving protocols proposed in Section 5.3 and Section 5.4, it is required the computation of the success probability in a network where the value of *L* is not unique for all the nodes in the route. For instance, consider Figure A1 with N=3 and p=0.5, define L=4 for the first node $N_{1}^{\left[1,{\bar{u}}_{1}\right]}$ and L=∞ for the other nodes ($N_{2}^{\left[1,{\bar{u}}_{1}\right]}$, $N_{3}^{\left[1,{\bar{u}}_{1}\right]}$, $N_{4}^{\left[1,{\bar{u}}_{1}\right]}$). The probability of successful transmission between the first and last nodes can be calculated by implementing a cluster in the network, that is, splitting the transmission of the packet from the source to the sink by a set of hops with the same value of *L*. In the example, $N_{1,2}=1$ denotes a sub-network composed by a unique hop between $N_{1}^{\left[1,{\bar{u}}_{1}\right]}$ to $N_{2}^{\left[1,{\bar{u}}_{1}\right]}$, while $N_{2,4}=2$ represents a sub-network composed by two hops between $N_{2}^{\left[1,{\bar{u}}_{1}\right]}$ to $N_{4}^{\left[1,{\bar{u}}_{1}\right]}$, such that the success probability for the route connecting the source and the sink is given by

$$P_{S}={P_{S}}_{1,2}\left(p=0.5,N=N_{1,2}=1,L=4\right)\times {P_{S}}_{2,4}\left(p=0.5,N=N_{2,4}=2,L=\infty \right).$$

Note that ${P_{S}}_{1,2}\left(p=0.5,N=N_{1,2}=1,L=4\right)$ and ${P_{S}}_{2,4}\left(p=0.5,N=N_{2,4}=2,L=\infty \right)$ can be computed by the R-package by using, respectively, the following commands:

> HBH(0.5, 0.5, 4 , 1)

and

> HBH(0.5, 0.5, Inf , 2).

Analogously, the first sub-net has an expected number of transmissions given by $E{\left(M\right)}_{1,2}$, while the second half expected number of transmissions is denoted by $E{\left(M\right)}_{2,4}$. The value for the entire network is given by

$$E\left(M\right)=E{\left(M\right)}_{1,2}+{P_{S}}_{1,2}\times E{\left(M\right)}_{2,4},$$

where the value of the expected number of transmission in the second half is weighted by the probability of the packet reaching this subnet (${P_{S}}_{1,2}$).

Using the procedure described above, it is possible to determine the values presented in Table 6 and Table 7 of the paper.
